# Nuclear-mitochondrial DNA segments resemble paternally inherited mitochondrial DNA in humans

**DOI:** 10.1038/s41467-020-15336-3

**Published:** 2020-04-08

**Authors:** Wei Wei, Alistair T. Pagnamenta, Nicholas Gleadall, Alba Sanchis-Juan, Jonathan Stephens, John Broxholme, Salih Tuna, Christopher A. Odhams, Genomics England Research Consortium, J. C. Ambrose, E. L. Baple, M. Bleda, F. Boardman-Pretty, J. M. Boissiere, C. R. Boustred, M. J. Caulfield, G. C. Chan, C. E. H. Craig, L. C. Daugherty, A. de Burca, A. Devereau, G. Elgar, R. E. Foulger, T. Fowler, P. Furió-Tarí, J. M. Hackett, D. Halai, J. E. Holman, T. J. P. Hubbard, R. Jackson, D. Kasperaviciute, M. Kayikci, L. Lahnstein, K. Lawson, S. E. A. Leigh, I. U. S. Leong, F. J. Lopez, F. Maleady-Crowe, J. Mason, E. M. McDonagh, L. Moutsianas, M. Mueller, N. Murugaesu, A. C. Need, C. A. Odhams, C. Patch, D. Perez-Gil, D. Polychronopoulos, J. Pullinger, T. Rahim, A. Rendon, P. Riesgo-Ferreiro, T. Rogers, M. Ryten, K. Savage, K. Sawant, R. H. Scott, A. Siddiq, A. Sieghart, D. Smedley, K. R. Smith, A. Sosinsky, W. Spooner, H. E. Stevens, A. Stuckey, R. Sultana, E. R. A. Thomas, S. R. Thompson, C. Tregidgo, A. Tucci, E. Walsh, S. A. Watters, M. J. Welland, E. Williams, K. Witkowska, S. M. Wood, M. Zarowiecki, NIHR BioResource, Alba Sanchis-Juan, Jonathan Stephens, Salih Tuna, Ernest Turro, Patrick F. Chinnery, Carl Fratter, Ernest Turro, Mark J. Caulfield, Jenny C. Taylor, Shamima Rahman, Patrick F. Chinnery

**Affiliations:** 1 Department of Clinical Neurosciences, School of Clinical Medicine, University of Cambridge, Cambridge Biomedical Campus, Cambridge, CB2 0QQ UK; 2 Medical Research Council Mitochondrial Biology Unit, University of Cambridge, Cambridge Biomedical Campus, Cambridge, CB2 0QQ UK; 3 Wellcome Centre for Human Genetics, University of Oxford, Oxford, OX3 7BN UK; 4 National Institute for Health Research (NIHR) Oxford Biomedical Research Centre, Oxford, OX3 7BN UK; 5 Department of Haematology, University of Cambridge, Cambridge Biomedical Campus, Cambridge, CB2 0AW UK; 6 Genomics England, London, UK; 7 Oxford Genetics Laboratories, Oxford University Hospitals NHS Foundation Trust, Churchill Hospital, Oxford, OX3 7LE UK; 8 Medical Research Council Biostatistics Unit, Cambridge Institute of Public Health, University of Cambridge, Cambridge, CB2 0SR UK; 9 William Harvey Research Institute, Queen Mary University of London, London, EC1M 6BQ UK; 10 Metabolic Department, Great Ormond Street Hospital for Children NHS Foundation Trust, London, WC1N 3JH UK; 11 UCL Great Ormond Street Institute of Child Health, London, WC1N 1EH UK

**Keywords:** Eukaryote, Genetic variation, Mitochondrial genome

## Abstract

Several strands of evidence question the dogma that human mitochondrial DNA (mtDNA) is inherited exclusively down the maternal line, most recently in three families where several individuals harbored a ‘heteroplasmic haplotype’ consistent with biparental transmission. Here we report a similar genetic signature in 7 of 11,035 trios, with allelic fractions of 5–25%, implying biparental inheritance of mtDNA in 0.06% of offspring. However, analysing the nuclear whole genome sequence, we observe likely large rare or unique nuclear-mitochondrial DNA segments (mega-NUMTs) transmitted from the father in all 7 families. Independently detecting mega-NUMTs in 0.13% of fathers, we see autosomal transmission of the haplotype. Finally, we show the haplotype allele fraction can be explained by complex concatenated mtDNA-derived sequences rearranged within the nuclear genome. We conclude that rare cryptic mega-NUMTs can resemble paternally mtDNA heteroplasmy, but find no evidence of paternal transmission of mtDNA in humans.

## Abstract (web summary)

Recent evidence has questioned the dogma of strict maternal transmission of mitochondrial DNA (mtDNA) in humans. Wei et al. saw no evidence of paternal transmission of mtDNA in 11,035 human trios, and show that nuclear-mitochondrial segments (NUMTs) can give the impression of paternal mtDNA transmission, but are actually inherited through the nuclear genome.

## Introduction

Mitochondrial DNA (mtDNA) is exclusively inherited down the maternal line in most eukaryotes^1^. From an evolutionary perspective, this probably evolved to suppress the presence of a mixed species of mtDNA (heteroplasmy) within cells, which can be disadvantageous^2^. Male and female gametes differ markedly in their mtDNA content, with oocytes typically containing >100–1000 fold more mtDNA molecules than sperm^3^, implying a simple mechanism where sperm mtDNA is simply ‘diluted out’ after fertilization. However, ultra-deep sequencing of informative human pedigrees does not support this hypothesis^3^, in keeping with an active process of destroying sperm mitochondria after fertilization^4^.

Despite these findings, the observation of rare mtDNA haplotypes that could have arisen through inter-molecular recombination^5^ raises the possibility of paternal mtDNA transmission at some point in the past. The human data are supported by observations in other vertebrates (*Ovis aries*^6^, *Parus major*^7^), but in most mammals the “leakage” of paternal mtDNA during transmission is seen in highly unusual situations, such as inter-species breeding in mice^8^, in vitro embryo manipulation in cattle (*Bos taurus*)^9^, or once in a rare human mitochondrial disease^10^. Two surveys of patients with mtDNA disorders failed to identify any additional cases of paternal mtDNA transmission, leading some to question the earlier findings^11,12^. However, the description of three large families reported to have biparental inheritance of mtDNA^13^ has rekindled the debate^14,15^. Paternal inheritance of mtDNA could have implications for forensic science, anthropology, and the genetic counselling of mtDNA diseases which affect ~1 in 5000^16^, so determining how frequently paternal transmission occurs is an important issue to resolve. To address this, we searched for the signature of biparental mtDNA inheritance in 33,105 whole genome sequences (WGS). We show that rare inherited nuclear-encoded mitochondrial segments (NUMTs) can create the impression of heteroplasmy resembling the signature of paternally transmitted mtDNA.

## Results and discussion

### Detecting mixed haplotypes

**Fig. 1:**
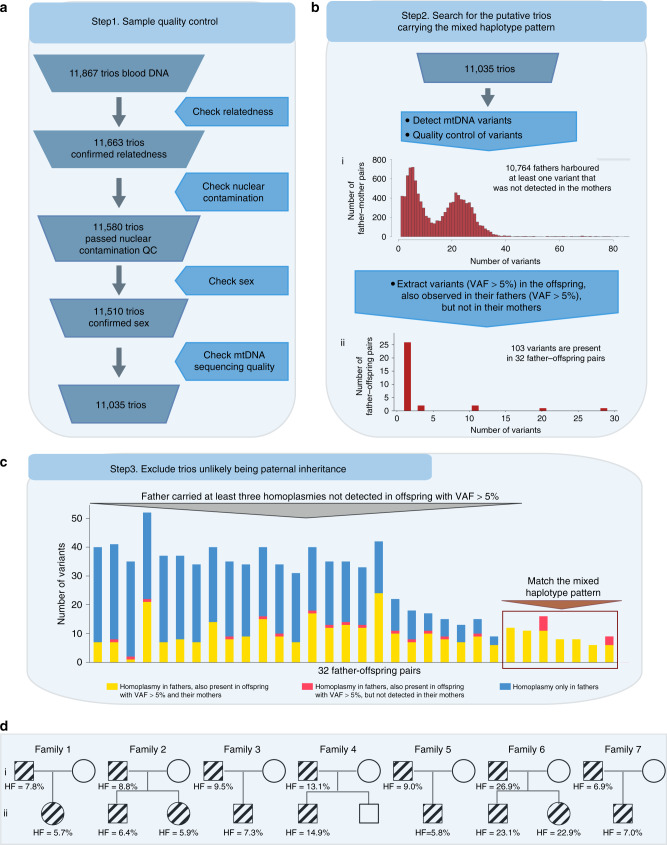
Bioinformatic pipeline to detect the mixed haplotypes from 11,867 trios. **a** Sample quality control. **b** Search for the putative trios carrying the mixed haplotype pattern. I. Distribution of the informative trios where at least one variant (variant allele fraction (VAF) > 5%) is detected in the father, but not in the mother. II. Distribution of trios where at least one variant was shared in the fathers and their offspring. **c** Number of homoplasmic variants in 32 fathers. Yellow bars represent fathers’ homoplasmic variants also observed in the mothers and their offspring; pink bars represent the homoplasmic variants also observed in their offspring, but not detected in the mothers. Blue bars represent homoplasmic variants only observed in the fathers which are not detected in either the mothers or their offspring. **d** Pedigrees of the seven families showing mixed haplotypes. The symbols with lines represent the individuals carrying the mixed haplotypes.

**Fig. 2:**
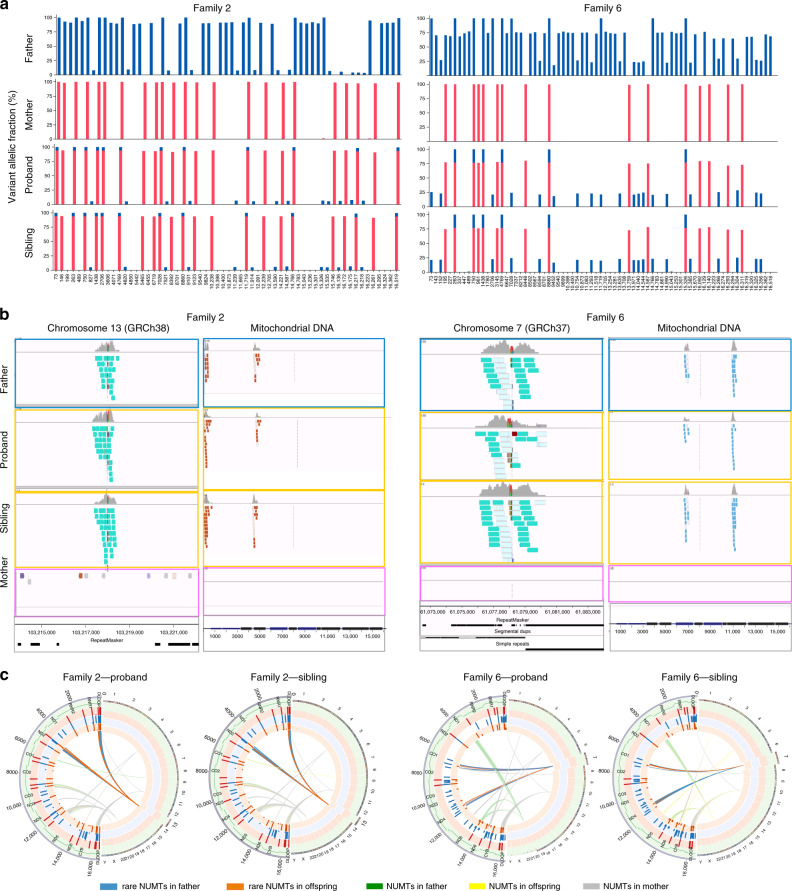
Mixed haplotype patterns and NUMTs observed in two families. **a** Examples of the mixed haplotype patterns showing Family 2 and Family 6. Father and both offspring show the mixed haplotypes with the similar HFs in each family. The mixed haplotype patterns observed in the other five families are shown in Supplementary Fig. 2. **b** Screenshots from Integrative Genomics Viewer (IGV) showing aligned reads corresponding to the rare NUMTs. The alignment of the discordant and split reads corresponding to the NUMTs on the nuclear DNA (left) and mtDNA (right) in two families. Teal bars indicate the aligned reads which mapped to the nuclear DNA where their mates mapped to the mtDNA (left). The chromosomes on which their mates are found are shown in different colours (right). The genome position, repeats and segmental duplications tracks from UCSC genome browser are shown at the bottom. IGV Screenshots from all aligned reads corresponding to the rare NUMTs on the nuclear DNA are shown in Supplementary Fig. 3. IGV screenshots of the other five families are shown in Supplementary Fig. 4. **c** Circos plots show the observed variants and NUMTs in two families. Circles from the outside to the inside indicate the following: (1) position of a variant on the mtDNA; (2) regions corresponding to the different mtDNA genes; (3) variants identified in the mother where the radial axis corresponds to the VAF; (4) variants identified in the father; (5) variants identified in the offspring, proband (left) and sibling (right) are shown, respectively; (6) NUMTs observed in the family, proband (left) and sibling (right) are shown, respectively.

After quality control (QC) steps, 33,105 individuals, including 11,035 unrelated mother-father-trios, were identified and included in this study from 35,601 WGS (mean depth = 42×, range from 30× to 99×) (Fig. 1a and Supplementary Fig. 1, for extensive QC see Methods). MtDNA-aligned variants were called using an established pipeline^17^. To increase the specificity we increased the threshold for the allele fraction (AF) to 5% in this analysis (Methods). We identified 10,764 trios where the father harboured at least one variant (AF > 5%) that was not detected in the mother, making the trio informative (Fig. 1bi). Next, we searched for the trios where at least one variant was shared by both the father and child, and the same variant was not detected in the mother. This defined 103 informative variants present in 32 children and their fathers which were not detected in their mothers (Fig. 1bii). If there was paternal transmission observed in these 32 father–offspring pairs, then all of the homoplasmic mtDNA variants (AF > 95%) in father should also be detectable in the offspring, and not just the some of them. Based on this, we excluded 25 out of 32 trios where the father carried at least three homoplasmic variants that were not observed in their offspring (Fig. 1c, Methods). This left seven trios harbouring mixed haplotypes bearing a striking resemblance to the observations made in the families reported to have biparental transmission of mtDNA^13^ (Figs. 1d, 2a and Supplementary Fig. 2). In three there were more than one offspring (Fig. 1d), with siblings from Family 2 and Family 6 having the same mixed haplotype as the probands and fathers (Fig. 2a). In Family 4, the haplotype was observed in one child (at ~15% AF) but not in the sibling (Supplementary Fig. 2).

On face value, these observations indicate that mixed haplotypes suggestive of possible paternal mtDNA transmission are found in ~0.06% of families. Although rare, this is more common than previously thought^10^. It should be noted that this percentage was derived using a specific filtering strategy. However, relaxing the criteria did not affect our overall conclusion. If correct, these observations have profound implications for our understanding of mtDNA evolution^5^, and the transmission of mtDNA diseases. We therefore set out to exclude alternative explanations, including the possibility that the paternally transmitted haplotypes were due to nuclear-mitochondrial DNA segments (NUMTs) embedded within the nuclear genome. NUMTs are ultimately derived from mtDNA in a distant ancestor^18^, but are transmitted autosomally.

### Detection of NUMTs

Analyzing 33,105 whole nuclear genome sequences from 11,035 trios (Methods), we found that all seven father–offspring pairs carried at least one novel NUMT with two breakpoints on the mtDNA sequence more than 500 bp away from each other (Fig. 2b, c, Table 1, Supplementary Figs. 3 and 4). These NUMTs have not been seen previously^19,20^, and were extremely rare in our dataset (<0.018%, the most common NUMT shared by 6 individuals from 3 unrelated families, Table 1). The same NUMTs were not observed in any of the seven mothers, nor in the second sibling in Family 4 (Fig. 2b, c, Table 1, Supplementary Figs. 3 and 4). None of the NUMTs disrupted the coding region, and mitochondrial disease was not suspected in any of the families (Fig. 2b, c, Supplementary Figs. 3 and 4). Four of the seven NUMTs were in genomic regions known to harbour repeat sequences and/or segmental duplications, as seen before^21^ (Fig. 2b, Supplementary Figs. 3 and 4).

**Table 1:** Summary of transmitted mega-NUMTs in eight families.

Family ID	Junction1 (hg38)		Junction2 (hg38)		No.	Father		Proband		Sibling		Mother
	Nuclear	mtDNA	Nuclear	mtDNA		DCRs	SPRs	DCRs	SPRs	DCRs	SPRs	
Family 1	chr17:76460877	1641(+)	chr17:76460893	13441(−)	2	37	29	40	14	NA	NA	None
Family 2	chr13:103216861	63(+)	chr13:103216872	4555(−)	3	15	3	34	6	17	10	None
Family 3	chr12:58832359	5523(+)	chr12:58832360	16109(+)	2	26	14	26	11	NA	NA	None
Family 4	chr7:61095411	11198(+)	chr7:61095402	6793(−)	5	9	10	8	4	None	None	None
Family 5	chr3:56128996	11126(+)	chr3:56128997	3128(−)	6	23	15	26	12	NA	NA	None
Family 6	chr7:61095411	11198(+)	chr7:61095402	6793(−)	5	10	3	8	4	18	1	None
Family 7	chr3:56128996	11126(+)	chr3:56128997	3128(−)	6	32	7	26	16	NA	NA	None
Family 8	chr3:176531354	247(−)	chr3:176531398	16405(+)	2	42	18	26	17	NA	NA	None

*No*. Number of individuals carrying the mega-NUMTs in the whole dataset, *DCRs* discordant reads, *SPRs* split reads, *None* not present, *NA* sample not available.

Despite their rarity of the NUMTs, Family 5 and Family 7, shared the same mixed haplotype and an identical NUMT, which was transmitted from father to offspring but was not detected in either mother (Table 1 and Supplementary Fig. 4). The same NUMT and mixed haplotypes were also observed in one mother in a different family, and was transmitted to her child, with both showing the same haplotype AF (Supplementary Fig. 5). These families were not known to be related, adding weight to the argument that the mixed haplotype in these families is due to the inheritance of a rare NUMT. Family 4 and Family 6 also shared an identical NUMT transmitted from father to two offspring in Family 6, but only detected in one child in Family 4 (Fig. 2b, c, Table 1, Supplementary Fig. 3 and Supplementary Fig. 4). Interestingly, in Family 4, the mother and proband also shared a different unique NUMT on chromosome 5. The second sibling did not inherit any of these NUMTs, so the mixed haplotypes were observed in the father, mother and first sibling, but not in the second sibling (Supplementary Figs. 2 and 4).

### Inheritance of the NUMTs is consistent with autosomal transmission

Next, we took an alternative approach, returning to the whole data set to search for all of the fathers who had NUMTs (frequency < 0.1% in our dataset, with the distance between two breakpoints being >500 bp further from each other on the mtDNA sequence) by identifying men with more than 12 heteroplasmic variants (AF > 1%) (Methods). This identified 14 fathers harbouring NUMTs, including all 7 families originally identified through the offspring, an additional father–offspring pair where the mixed haplotype was transmitted from father to offspring with an AF < 5% (and thus was excluded from our original analysis based on the low AF) (Family 8 in Table 1) (Supplementary Fig. 6). In the other 6 fathers, the mixed haplotype was not detected in the offspring (Supplementary Fig. 7 and Supplementary Table 1). All the NUMTs from those individuals were confirmed by both the discordant and split reads (Supplementary Fig. 7 and Supplementary Table 1). Overall, the proportion of these NUMTs transmitted was 58.8% (In 10 of 17 father–offspring pairs from 14 unrelated families, fathers transmitted the rare NUMTs to their offspring, Clopper-Pearson 95% CI = 32.9–81.6), consistent with autosomal transmission.

### Detecting multiple fragments of mtDNA within the NUMTs

**Fig. 3:**
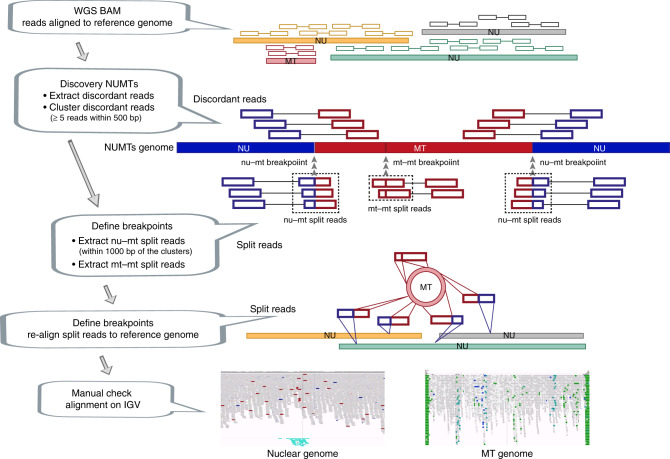
Strategy for NUMT detection. NU – nuclear genome, MT – mitochondrial genome. nu-mt split reads: one end of the split read maps to nuclear DNA and the other end maps to mtDNA-derived sequence. mt-mt split reads: two ends of the same split read map to two locations on the mtDNA-derived sequences. nu-mt breakpoint: the breakpoint between joined nuclear and mtDNA-derived sequences. mt-mt breakpoint: the breakpoint joint two separate mtDNA-derived sequences. For a detailed explanation see Methods.

**Fig. 4:**
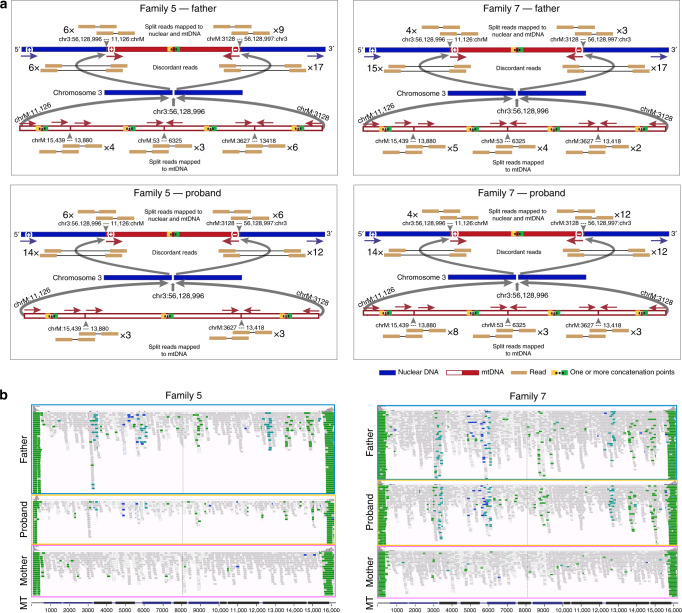
Defining complex mega-NUMTs. **a** Split reads align to both mtDNA-derived and nuclear DNA sequences (top). Discordant reads are paired reads where one end aligns to mtDNA-derived sequences and the other end aligns to nuclear DNA sequences (middle). Possible constructed concatemer is shown at the bottom with observed supporting split reads. The positions of breakpoints (bp) are shown on both nuclear DNA (top) and mtDNA (bottom). **b** IGV screenshots showing the reads not properly aligned to mtDNA-derived sequences. The reads are coloured by pair orientation. Many read pairs with anomalous pair orientations in the fathers and probands support the mtDNA-derived sequence rearrangement. In Family 5 and Family 7, the fathers and offspring carried the same NUMT, they also carried the same nuclear-mtDNA junctions and the junctions within mtDNA-derived sequences. The defined junctions by split reads from the other five families are included in Supplementary Fig. 8.

**Fig. 5:**
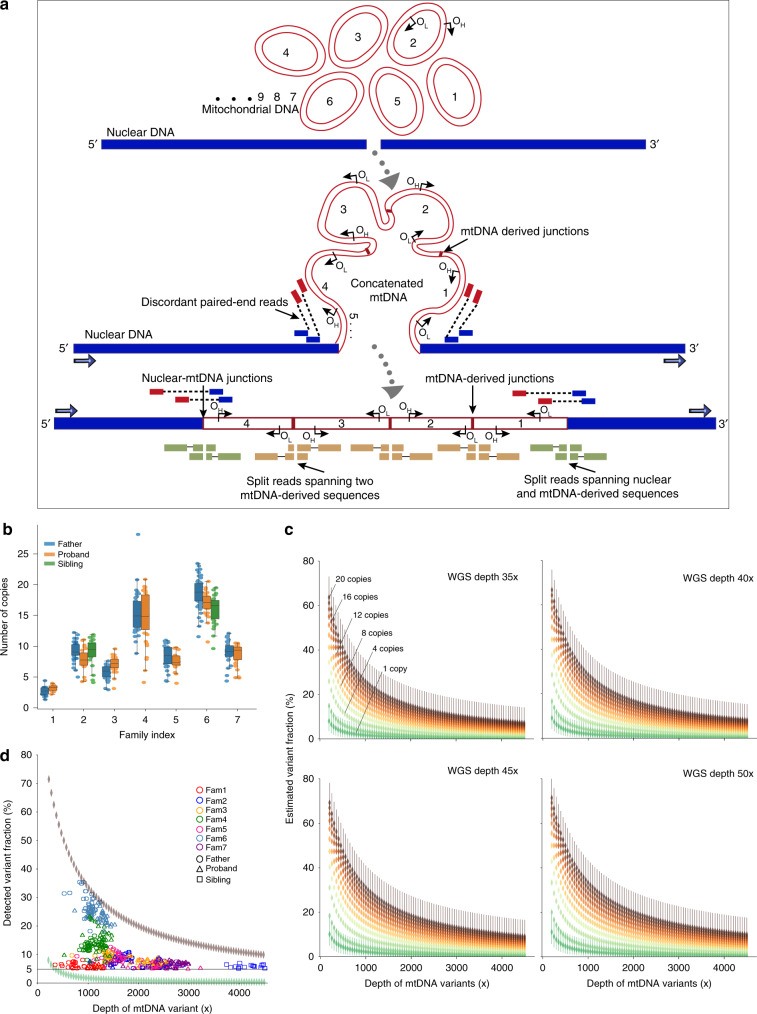
Characteristics of mega-NUMTs. **a** Model showing the formation of mega-NUMTs and our strategy for their detection in whole genome sequence data. OH_,_ origins of heavy-strand replication; OL_,_ origins of light-strand replication. **b** Combined box and swarm plots show the estimated number of copies of the mtDNA-derived fragment within the NUMT in seven families. The middle “box” represents the median, lower and upper quartile of the data. The upper and lower whiskers represent the data outside the middle 50%. The dots represent the informative variants included in the mixed haplotype (Methods, Supplementary Table 4). **c** Modelling of the estimated variant allelic fraction for a NUMT at different true mtDNA sequencing depths. Modelling was based on whole genome nuclear sequencing (WGS) depths seen in our dataset (35×, 40×, 45× and 50×), and the corresponding variant fraction based on the number of copies of mtDNA-derived fragment within the NUMT. 95% confident intervals are shown for one copy and 20 copies. **d** Detected variant allelic fraction for each of the seven families related to the true mtDNA per-base sequence depth. Upper and lower symbols show the trend of variant allelic fraction estimated from WGS depth 50× with 20 copies of mtDNA-derived fragments and WGS depth 35× with 1 copy of mtDNA-derived fragment.

Returning to the seven original families, we mapped the discordant reads to the mitochondrial genome. In each family, we saw that some of the alleles defining the mixed haplotype fell outside the minimum predicted size of the NUMTs (Fig. 2c, Supplementary Fig. 4). Importantly, all of the alleles within the mixed haplotype had a similar AF, irrespective of whether or not they fell within the smallest predicted NUMT. It should be noted that our stringent filters prevented the detection of some NUMTs in the genome. If present, these additional NUMTs would add further weight to our conclusions. To identify the breakpoints of each NUMT, we searched for the split reads (Fig. 3) (Methods). In six of the seven families the split reads mapped in opposite directions at the two ends of the same NUMT. We were unable to find any other nuclear structural variations in the surrounding region to explain this observation, indicating a complex rearrangement not just involving the smallest predicted NUMT (Fig. 4, Table 1 and Supplementary Fig. 8). In addition, all seven families had at least one unique junction supported by more than two split reads mapping to two different parts of the mtDNA-derived sequence (Fig. 4, Supplementary Fig. 8 and Supplementary Table 2). These observations pointed towards the existence of very large NUMTs (mega-NUMTs) containing multiple concatenated copies of the mtDNA-derived sequence within the boundaries defined by the split reads, as seen other species^18,22^ (Fig. 5a). Several lines of evidence in support this. First, all individuals with the same mega-NUMT also had the same mtDNA-derived junctions (Fig. 4, Supplementary Fig. 8 and Supplementary Table 2). Second, we never found more than two split reads supporting any unique mtDNA-derived junctions in the family members who did not have the mixed haplotype. Third, mtDNA-derived junctions detected in the fathers who carried non-transmitted mega-NUMTs were never seen in their offspring (Supplementary Fig. 7 and Supplementary Table 2). Thus, the rare mtDNA-derived junctions co-segregate with the mega-NUMT in multiple unrelated families, implying that they are structurally related to the mega-NUMT within the nuclear genome. Given that long tandem repeats predispose to structural genomic variants^23^, it is likely these intra-mtDNA rearrangements occurred following the original NUMT integration event^24^.

### Estimating the number of mtDNA fragments within each NUMT

Finally, if the mixed haplotype was encoded by the nuclear genome, then the AFs should decrease when the amount of mtDNA increased. To explore this, we harnessed ~3-fold difference in whole-blood mtDNA content arising from natural fluctuations in blood cell composition^25^. First, the number of copies of mtDNA-derived fragments within NUMTs was estimated to be between 2 and 20 (Fig. 5b, Methods). Importantly, the father and offspring from the same family carried a similar number of copies of the mtDNA-derived fragment; and families carrying the same NUMT had a similar number the mtDNA-derived fragments. Next, we modelled the theoretical haplotype AF for a NUMT with increasing mtDNA sequence coverage, scaling this upwards for sequences present more than once in the nuclear genome (NUMTs) (Fig. 5c). As predicted, higher mtDNA content was inversely correlated with the haplotype AF (*R*^2^ = −0.53, *P* < 2.2 × 10^−16^), and the same trajectory was seen for individuals within the same family (Fig. 5d).

### Validation using long-read sequencing

**Fig. 6:**
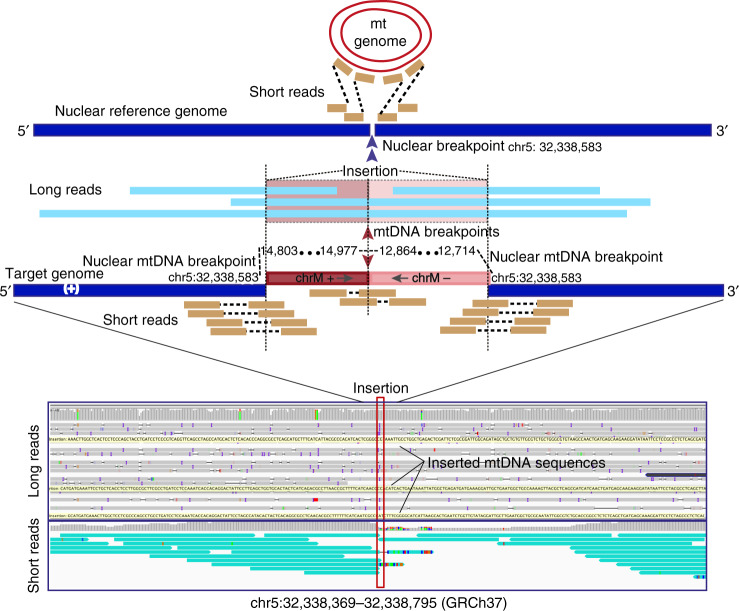
Validation of NUMTs using long-read (Oxford Nanopore PromethION) sequencing. Example shown is a concatenated mtDNA NUMT detected by both long-read sequencing and short-read sequencing. mtDNA and nuclear genome reference sequences are shown on the top in red and navy. Aligned short reads shown in orange and long reads in light blue. The sequenced genome included concatenated mtDNA sequences (fragment 1: mt 14803-14977 (+) and fragment 2: 12864-12714 (−)) inserted into chr5: 32,338,583. The two mtDNA fragments are coloured in red and plink. The sequences of the long reads aligned to two mtDNA fragments are also highlighted in red and pink. The remaining sequences aligned to nuclear genome in the same region. The IGV screenshot shows both long-read (top) and short-read (bottom) alignments. The insertion point on the nuclear genome is highlighted by the red box. The yellow bars on the long-read alignment are the large insertions from mtDNA sequences. Teal bars on the short-reads alignment are the discordant reads which one read aligned to nuclear genome and their mate reads aligned to mtDNA genome.

To validate our bioinformatic strategy for NUMT detection in short-read sequencing, we carried out long-read (Oxford Nanopore PromethION) whole genome sequencing (WGS) in five individuals from the NIHR BioResource - Rare Diseases project^26^ (Methods), where short-read WGS data was also available from the same individuals^26^. Twenty-three NUMTs were detected from five individuals using short-read WGS. In the long-read sequencing data, all 23 NUMTs were supported by aligned long reads covering the entire NUMT. Large insertions from mtDNA sequences were observed in the aligned reads (Fig. 6) (Supplementary Table 3) (Methods). Interestingly, we observed that a common NUMT present in three of five individuals (68% in 11,035 trios) contained two separate fragments of the mtDNA sequence (fragment 1: mt 14803-14977 (+) and fragment 2: 12864-12714 (−)) incorporating two fragments from different strands of mtDNA concatenated and inserted into nuclear genome (Fig. 6). This observation confirmed that concatenated mtDNA NUMTs exist in humans, and that they are a common finding.

In conclusion, our findings support the hypothesis^27^ that large rare NUMTs, or mega-NUMTs, can masquerade as a heteroplasmic haplotype, giving the impression of biparental transmission of mtDNA. Based on an analysis of 11,035 trios, we find no evidence to reject the established dogma that human mtDNA is exclusively inherited down the maternal line.

## Methods

### Study samples

We studied 35,601 WGS data from whole-blood DNA in the Genomics England 100,000 Genomes Rare Disease Main Programme^28^. DNA was extracted using Qiagen DNA extraction protocols and following quality assurance and quantification 4.5 µg of DNA was submitted to Illumina Inc at their Great Chesterford centre. After sample quality control (QC) (details below) (Fig. 1a), 11,035 trios were included in this study.

### Ethical approval

Ethical approval was provided by the East of England Cambridge South national research ethics committee under reference number: 13/EE/0325, with participants providing written informed consent for this approved study. All consenting participants in the Rare Disease arm of the 100,000 Genomes Project were enroled via thirteen centres in the National Health Service covering all NHS patients in England.

### Extracting mitochondrial sequences and detecting variants

Next generation sequencing of the whole genome from whole-blood DNA was performed on Illumina HiSeqX (Illumina, Inc., San Diego, CA, USA) according to standard operating procedures and using the bio‐informatics pipeline developed for the Genomics England Main Programme analysis^28^. Following quality assurance the short reads (150 bp) were aligned to the human genome builds (GRCh 37 and/or GRCh 38) using the ISAAC Genome Aligner with options: --bam-gzip-level 6 --cleanup-intermediary 1 --base-quality-cutoff 15 --gap-scoring bwa --variable-read-length yes --ignore-missing-bcls 1 --ignore-missing-filters 1 --split-gap-length 10000 --per-tile-tls 1 --seed-length 32 --barcode-mismatched 1 --use-bases-mask Y150N1,Y150N1 --base-calls-format bcl-gz for GRCh37, --bam-gzip-level 6 --scatter-repeats 1 --cleanup-intermediary 1 --base-quality-cutoff 15 --clip-semialigned 1 --gap-scoring bwa --variable-read-length yes --ignore-missing-bcls 1 --ignore-missing-filters 1 --split-gap-length 10000 --seed-length 16 --barcode-mismatched 1 -use-bases-mask Y150N1,Y150N1 --base-calls-format bcl-gz/fastq-gz for GRCh38, and the BAM files were generated. The mean depth of WGS was 42× (range from 30× to 99×) (Supplementary Fig. 1). The subset of sequencing reads which aligned to the mitochondrial genome were extracted from each WGS BAM file. MtDNA sequences were processed using an established pipeline^17^. We ran MToolBox (v1.0) on the resulting smaller BAM files to generate the realigned mtDNA BAM files^29^. The realigned bam files were used to call the variants. We then filtered the variants as follows: (1) retaining variants for which the allele fractions (AFs) were above 1%; (2) retaining only single nucleotide polymorphisms (SNPs); (3) removing variants with depth < 200×; (4) removing variants <2 reads on each strand for the minor allele; (5) remove variants falling within low-complexity regions (66–71, 300–316, 513–525, 3106–3107, 12418–12425 and 16182–16194).

mtDNA haplogroup assignment was performed using HaploGrep2^30,31^.

### Quality control of samples

We estimated the degree of relatedness between individuals using an established pipeline^17^. Briefly, a list of 32,665 autosomal SNPs was selected to estimate relatedness. By filtering the merged VCF and the 1000 G reference set with the selected SNPs, pc-relate function from the GENESIS package^32^ was applied to obtain the pairwise relatedness. First 20 principal components were used to weight the population structure. Reference set was used to increase genetic diversity accounted for by the PCA. Two hundred and four of 11,867 trios were excluded in this study because the father and/or mother relatedness could not be confirmed by the genomic data.

Potential DNA cross-contamination was investigated using the nuclear genome. All samples passed contamination quality checks conducted by the sequencing provider Illumina, Inc. Additionally, we estimated the degree to which a DNA sample was contaminated by any other DNA sample using verifyBamID^33^. Eighty-three samples with an estimate of contamination (FREEMIX) exceeding 3% were excluded in this study. To further check for possible contamination of the seven families carrying the mixed haplotypes, we calculated the number of extreme heterozygotes with AF beyond the range of 25–75% in each individual from seven families (Supplementary Fig. 9) using the remaining individuals from the whole dataset as controls. All seven families carried very few extreme heterozygotes making it unlikely that there was sample contamination.

Next, we determined sex by comparing the average depth of sex chromosomes. If the average depth of chromosome X was 10 times greater than the average depth of chromosome Y, then the sample was defined as female. We excluded 70 trios where father and/or mother’s sex was inconsistent with the recorded sex.

Finally, we removed the trios where the average depth of mtDNA from one family member was below 500×. After all the sample QC steps, 11,035 trios were included in the final analysis.

### Searching for the putative trios carrying the mixed haplotypes

We searched for the same mtDNA biparental inheritance pattern reported by Luo et al.^13^, looking for potentially paternally transmitted alleles present at AF > 5% in the offspring in 11,035 trios (note, in each case, Luo et al.^13^ observed AF > 20% in the offspring). First, we counted the number of informative trios where the father harboured at least one variant (AF > 5%) that was not detected in the mother. If the father shared a variant with the mother, this was considered non-informative. Figure 1bi shows the distribution of trios where at least one variant was detected in the father and not in the mother. The left peak in Fig. 1bi includes father–mother pairs from the same mtDNA haplogroup background. The right peak includes father–mother pairs from two different mtDNA backgrounds, hence the greater number of variants (Supplementary Fig. 10). Next, we extracted the trios where at least one variant was shared by both the father and child, and the same variant was not detected in the mother. This defined 103 informative variants present in 32 children and their fathers that were not detected in their mothers (Fig. 1c). If there was paternal transmission observed in these 32 father–offspring pairs, then all the homoplasmic mtDNA variants in father should be detectable in the offspring, and not just the some of them. Homoplasmy was conservatively defined as an AF of >95%. However, in 25 trios, the father carried at least three homoplasmic variants that were not observed in their offspring at AF > 5% (Fig. 1c), despite those fathers and their offspring sharing some variants which were not detected in the mothers. The absence of these variants made paternal transmission extremely unlikely, so these 25 trios were excluded from subsequent analysis.

### Detecting the NUMTs and breakpoints

To detect NUMTs, we used a modified approach described by Ju et al.^34^. From the aligned WGS bam files, we extracted the discordant read pairs using samblaster^35^, and remained the read pairs where one end aligns to nuclear genome and the other end aligns to the mtDNA reference sequence. The reads with mapping quality below 20 were discarded. The discordant reads were then clustered together based on sharing the same orientation and whether they were within a distance of 500 bp. We analyzed clusters supported by at least five pairs of discordant reads.

To identify putative breakpoints spanning nuclear DNA and a mtDNA-derived sequence, we searched for the split reads within a distance of 1000 bp of discordant reads which were then re-aligned using BLAT^36^. We further analyzed the re-aligned reads where one end of the read mapped to nuclear DNA and the other end of the same read mapped to mtDNA-derived sequence. To identify putative breakpoints spanning two locations on the mtDNA-derived sequence, we extracted the split reads which only aligned to mtDNA sequence. Those split reads were further re-aligned using BLAT. We analyzed the reads where the two ends of the same read mapped to two locations on the mtDNA sequence.

Because WGS were aligned to the human genome builds GRCh 37 and/or GRCh 38, to calculate the frequencies of the observed NUMTs in the full dataset, we lifted over the sequences from GRCh37 to GRCh38 using the liftOver tool from UCSC (https://genome.ucsc.edu/cgibin/hgLiftOver), if they were initially aligned to GRCh37. Clusters within a distance of 1000 bp on both nuclear DNA and mtDNA were grouped as the same NUMT.

### Validating the NUMTs using long-read sequencing

To validate our bioinformatic strategy for NUMTs detection in short-read sequencing, we carried out WGS on Oxford Nanopore PromethION in five individuals from the NIHR BioResource - Rare Diseases project^26^. Long-read sequencing was performed on genomic DNA using the Oxford Nanopore Technologies (ONT) PromethION platform (ONT, Oxford, United Kingdom). In brief, 1 μg of 20 ng/μl DNA was sheared to an average fragment length of 10,000 bp by spinning in a Covaris G-Tube (Covaris, Woburn, Massachusetts) at 6000 rpm using an Eppendorf 5415 R Microcentrifuge (Eppendorf, Hamburg, Germany). Sheared DNA was then prepared for sequencing using the ONT SQK-LSK109 library prep kit and protocol GDE_9063_v109_revQ_14Aug2019. Libraries, containing only one sample each, were loaded into independent FLO-PRO002 flow cells which were run using the default 48 h PromethION protocol. Base calling was done using Guppy v.3.2.6. Reads passing QC during base calling were aligned to either GRCh37 or hg38 using minimap v.2.16-r922 and alignments processed using Samtools v1.9. The short-read WGS data from the same individuals are also available^26^. Firstly, we detected the NUMTs from short-reads WGS using the same pipeline as described above. We then extracted the reads aligned to the same region from long-read sequencing data in the same individual. The extracted reads were re-aligned using BLAT. All the observed NUMTs were also manually inspected on IGV^37^.

### Estimating the number of mtDNA fragments within each NUMT

The number of copies of mtDNA-derived fragments (Nmt) within the same NUMT was estimated as:

$${Nmt} = \frac{Altmt}{{DPadjnumt} \div 2}$$

where *DPadjnumt* is the average depth of the nuclear genome sequencing flanking the NUMT (derived from both complementary chromosomes); and *Altmt* is the number of reads supporting the alternative allele from the informative variants within the mixed haplotype. If the AF > 50%, Altmt = DPmtvar – Altmt’. DPmtvar is the depth of the informative variant, Altmt’ is the initial number of reads supported alternative allele.

### Estimating the mixed haplotype fractions

Given the sequence depth of both the nuclear DNA and true mtDNA, we estimated the mixed haplotype fractions (HTFs) based on different number of copies of mtDNA-derived fragments within a NUMT over the observed range of nuclear and mtDNA coverage within our dataset (Supplementary Fig. 1, nuclear genome depths: 35×, 40×, 45× and 50×; and true mtDNA sequence depth 200× to 4500×). The number of copies of mtDNA-derived fragments within the NUMTs were estimated at 1 copy to 20 copies. The mixed haplotype fraction was calculated as:

$${\mathrm{HTF}} = \frac{{\mathrm{DPnu} \div 2 \times \mathrm{Nmt}}}{{\mathrm{DPnu} \div 2 \times \mathrm{Nmt} + \mathrm{DPmt}}}$$

where DPnu is the depth of nuclear genome (35×, 40×, 45× and 50×); DPmt is the depth of true mtDNA variants (from 200× to 4500×); Nmt is the estimated number of copies of mtDNA-derived fragments within the same NUMT; and HTF is the estimated mixed haplotype fraction.

### Searching for paternally transmitted and non-transmitted NUMTs

We applied an independent pipeline to search for other fathers carrying both rare NUMTs and the mixed haplotypes. We identified fathers: (1) carrying more than 12 heteroplasmies with AF > 1% (interquartile range method to define the outliers) (Supplementary Fig. 11); and (2) carrying at least one large NUMT (with the distance between two breakpoints being >500 bp further from each other on the mtDNA sequence) which was rare in the whole dataset (frequency < 0.1%).

### Statistical analysis

All statistical analyses in this study were suggested in the text and performed using R (http://CRAN.R-project.org/). Figures were generated using Matplotlib (https://matplotlib.org) in Python (http://www.python.org) and R. Circos plots were made using Circos^38^.

### Reporting summary

Further information on research design is available in the Nature Research Reporting Summary linked to this article.

## Supplementary information

Supplementary Information

Reporting Summary

## Data Availability

The whole genome sequence data analysed in this study can be accessed through the Genomics England data warehouse https://www.genomicsengland.co.uk/understanding-genomics/data/. Researchers can apply for access to the data to reproduce our findings, or to carry out other analyses through the Genomics England Clinical Interpretation Partnerships (GeCIPs). The authors declare that all data supporting the findings of this study are available within the paper and its supplementary information files.
