# Author Correction: Nuclear-mitochondrial DNA segments resemble paternally inherited mitochondrial DNA in humans

**DOI:** 10.1038/s41467-020-17572-z

**Published:** 2020-07-22

**Authors:** Wei Wei, Alistair T. Pagnamenta, Nicholas Gleadall, Alba Sanchis-Juan, Jonathan Stephens, John Broxholme, Salih Tuna, Christopher A. Odhams, Genomics England Research Consortium, J. C. Ambrose, E. L. Baple, M. Bleda, F. Boardman-Pretty, J. M. Boissiere, C. R. Boustred, M. J. Caulfield, G. C. Chan, C. E. H. Craig, L. C. Daugherty, A. de Burca, A. Devereau, G. Elgar, R. E. Foulger, T. Fowler, P. Furió-Tarí, J. M. Hackett, D. Halai, J. E. Holman, T. J. P. Hubbard, R. Jackson, D. Kasperaviciute, M. Kayikci, L. Lahnstein, K. Lawson, S. E. A. Leigh, I. U. S. Leong, F. J. Lopez, F. Maleady-Crowe, J. Mason, E. M. McDonagh, L. Moutsianas, M. Mueller, N. Murugaesu, A. C. Need, C. A. Odhams, C. Patch, D. Perez-Gil, D. Polychronopoulos, J. Pullinger, T. Rahim, A. Rendon, P. Riesgo-Ferreiro, T. Rogers, M. Ryten, K. Savage, K. Sawant, R. H. Scott, A. Siddiq, A. Sieghart, D. Smedley, K. R. Smith, A. Sosinsky, W. Spooner, H. E. Stevens, A. Stuckey, R. Sultana, E. R. A. Thomas, S. R. Thompson, C. Tregidgo, A. Tucci, E. Walsh, S. A. Watters, M. J. Welland, E. Williams, K. Witkowska, S. M. Wood, M. Zarowiecki, NIHR BioResource, Alba Sanchis-Juan, Jonathan Stephens, Salih Tuna, Ernest Turro, Patrick F. Chinnery, Carl Fratter, Ernest Turro, Mark J. Caulfield, Jenny C. Taylor, Shamima Rahman, Patrick F. Chinnery

**Affiliations:** 1 Department of Clinical Neurosciences, School of Clinical Medicine, University of Cambridge, Cambridge Biomedical Campus, Cambridge, CB2 0QQ UK; 2 Medical Research Council Mitochondrial Biology Unit, University of Cambridge, Cambridge Biomedical Campus, Cambridge, CB2 0QQ UK; 3 Wellcome Centre for Human Genetics, University of Oxford, Oxford, OX3 7BN UK; 4 National Institute for Health Research (NIHR) Oxford Biomedical Research Centre, Oxford, OX3 7BN UK; 5 Department of Haematology, University of Cambridge, Cambridge Biomedical Campus, Cambridge, CB2 0AW UK; 6 Genomics England, London, UK; 7 Oxford Genetics Laboratories, Oxford University Hospitals NHS Foundation Trust, Churchill Hospital, Oxford, OX3 7LE UK; 8 Medical Research Council Biostatistics Unit, Cambridge Institute of Public Health, University of Cambridge, Cambridge, CB2 0SR UK; 9 William Harvey Research Institute, Queen Mary University of London, London, EC1M 6BQ UK; 10 Metabolic Department, Great Ormond Street Hospital for Children NHS Foundation Trust, London, WC1N 3JH UK; 11 UCL Great Ormond Street Institute of Child Health, London, WC1N 1EH UK

**Keywords:** Eukaryote, Genetic variation, Mitochondrial genome

Correction to: *Nature Communications* 10.1038/s41467-020-15336-3, published online 8 April 2020.

The original version of this Article contained an error in an equation in the Methods in the section “Estimating the number of mtDNA fragments within each NUMT”. The equation for calculating the number of mtDNA fragments within each nuclear-mitochondrial DNA segment and its explanation incorrectly read:

The number of copies of mtDNA-derived fragments (Nmt) within the same NUMT was estimated as:

$${\mathrm{Nmt}} = \frac{{{\mathrm{DPnumt}} \div 2}}{{{\mathrm{DPnumt}} \div 2 + {\mathrm{Altmt}}}}$$

where DPnumt is the average depth of the NUMT sequence surrounding region where discordant reads aligned on the nuclear genome; and Altmt is the number of reads supported alternative allele from the informative variants within the mixed haplotype. If the AF > 50%, Altmt = DPmt – Altmt’. DPmt is the depth of the informative variant, Altmt’ is the initial number of reads supported alternative allele.

The correct version of this paragraph is:

The number of copies of mtDNA-derived fragments (Nmt) within the same NUMT was estimated as:

$$Nmt = \frac{{Altmt}}{{DPadjnumt \div 2}}$$

where *DPadjnumt* is the average depth of the nuclear genome sequencing flanking the NUMT (derived from both complementary chromosomes); and *Altmt* is the number of reads supporting the alternative allele from the informative variants within the mixed haplotype. If the AF > 50%, Altmt = DPmtvar – Altmt’. DPmtvar is the depth of the informative variant, Altmt’ is the initial number of reads supported alternative allele.

We confirm that the correct equation was used in our analyses, and that this correction does not change our results.

This has now been corrected in the PDF and HTML versions of the Article.

