# Supplementary material for: Nuclear-mitochondrial DNA segments resemble paternally inherited mitochondrial DNA in humans
Source: Nat Commun. 2020 Apr 8;11:1740. doi: 10.1038/s41467-020-15336-3 (PMC7142097; doi:10.1038/s41467-020-15336-3)
Supplement: Supplementary file 1 — Supplementary Information [file 41467_2020_15336_MOESM1_ESM.pdf]

Supplementary Information for

Nuclear-mitochondrial DNA segments resemble paternally inherited mitochondrial DNA in humans

Wei *et al.*

Supplementary Note 1

NIHR BioResource Collaborators

NIHR BioResource - Rare Disease and Control Collections: NIHR BioResource Core Teams Anthony Attwood[3,4], Matthew Brown[3,4], Naomi Clements Brod[3,4], Abigail Crisp-Hihn[3,4], John Davis[3,4], Sri V V Deevi[3,4], Eleanor F Dewhurst[3,4], Karen Edwards[3,4], Marie Erwood[3,4], James Fox[3,4], Amy J Frary[3,4], Fengyuan Hu[3,4], Jennifer Jolley[3,4], Nathalie Kingston[4], Rachel Linger[4,43], Rutendo Mapeta[3,4], Jennifer Martin[4,22,43], Stuart Meacham[3,4], Sofia Papadia[4,43], Paula J Rayner-Matthews[3,4], Crina Samarghitean[3,4], Olga Shamardina[3,4], Ilenia Simeoni[3,4], Simon Staines[3,4], Emily Staples[22], Hannah Stark[4,43], Catherine Titterton[3,4], Julie von Ziegenweidt[3,4], Christopher Watt[3,4], Deborah Whitehorn[3,4], Yvette Wood[3,4], Katherine Yates[3,4,22], Ping Yu[3,4], Roger James[3,4], Sofie Ashford[4,43], Christopher J Penkett[3,4], Kathleen E Stirrups[3,4], John R Bradley[4,22,24,29,47]; **Bleeding, thrombotic and Platelet Disorders (BPD)** Tadbir Bariana[12,50], Claire Lentaigne[51,52], Suthesh Sivapalaratnam[45,53,54,55], Sarah K Westbury[56,57], David J Allsup[58,59], Tamam Bakchoul[60], Tina Biss[61], Sara Boyce[62], Janine Collins[3,53], Peter W Collins[63], Nicola S Curry[64], Kate Downes[3,4], Tina Dutt[65], Wendy N Erber[66], Gillian Evans[67], Tamara Everington[68,69], Remi Favier[70,71], Keith Gomez[12,50], Daniel Greene[3,4,49], Paolo Gresele[72], Daniel Hart[53], Rashid Kazmi[62], Anne M Kelly[24], Michele Lambert[73,74], Bella Madan[75], Sarah Mangles[69], Mary Mathias[76], Carolyn Millar[51,52], Samya Obaji[77], Kathelijne Peerlinck[78], Catherine Roughley[67], Sol Schulman[79], Marie Scully[80], Susan E Shapiro[64], Keith Sibson[76], Ilenia Simeoni[3,4], Matthew C Sims[3,81], R Campbell Tait[82], Kate Talks[61], Chantal Thys[78], Cheng-Hock Toh[65], Chris Van Geet[78], John-Paul Westwood[80], Sofia Papadia[4,43], Andrew D Mumford[56,57], Willem H Ouwehand[3,4,44,45,46], Kathleen Freson[78], Michael A Laffan[51,52]; **Cerebral Small Vessel Disease (CSVD)** Rhea Y Y Tan[30], Kirsty Harkness[83], Sarju Mehta[84], Keith W Muir[85], Ahamad Hassan[86], Matthew Traylor[30], Anna M Drazyk[30], Hugh S Markus[30]; **Ehlers Danlos Syndrome (EDS)** David Parry[7], Munaza Ahmed[87], Hanadi Kazkaz[80], Anthony M Vandersteen[88], Timothy J Aitman[6,7]; **Hypertrophic Cardiomyopathy (HCM)** Elizabeth Ormondroyd[36,37], Kate Thomson[36,37], Timothy Dent[37], Paul Brennan[15,16,17], Rachel J Buchan[89,90], Teofila Bueser[21,26,91], Gerald Carr-White[20], Stuart Cook[89,92,93,94], Matthew J Daniels[36,37,95], Andrew R Harper[35,36,89], James S Ware[89,90,92], Hugh Watkins[35,36,37]; **Intrahepatic Cholestasis of Pregnancy (ICP)** Peter H Dixon[41], Jenny Chambers[41,96], Floria Cheng[96], Maria C Estiu[97], William M Hague[98], Hanns-Ulrich Marschall[99], Marta Vazquez-Lopez[96], Catherine Williamson[41,42]; **Inherited Retinal Disorders (IRD)** Gavin Arno[38,39], Eleanor F Dewhurst[3,4], Marie Erwood[3,4], Courtney E French[100], Michel Michaelides[38], Anthony T Moore[38,39,101], Alba Sanchis-Juan[3,4], Keren Carss[3,4], Andrew R Webster[38,39], F Lucy Raymond[4,28]; **Leber Hereditary Optic Neuropathy (LHON)** Philip Griffiths[102,103], Rita Horvath[34,104], Gavin Hudson[34], Neringa Jurkute[12,38], Angela Pyle[34], Patrick Yu-Wai-Man[1,2,38,39]; **Multiple Primary Malignant Tumours (MPMT)** James Whitworth[28,29,105], Julian Adlard[106], Munaza Ahmed[87], Ruth Armstrong[28,29,105], Carole Brewer[107], Ruth Casey[28,29,105], Trevor R P Cole[108], Dafydd Gareth Evans[109], Lynn Greenhalgh[110], Helen L Hanson[111], Jonathan Hoffman[108], Louise Izatt[112], Ajith Kumar[87], Fiona Lalloo[14], Kai Ren Ong[108], Soo-Mi Park[29,105,113], Claire Searle[114], Lucy Side[87], Katie Snape[111], Emma Woodward[14], Marc Tischkowitz[113,115], Eamonn R Maher[28,29]; **Neurological and Developmental Disorders (NDD)** Keren Carss[3,4], Eleanor F Dewhurst[3,4], Marie Erwood[3,4], Courtney E French[100], Detelina Grozeva[28], Manju A Kurian[116,117], F Lucy Raymond[4,28]; **Neuropathic Pain Disorders (NPD)** Andreas C Themistocleous[10], David Gosal[118], Rita Horvath[34,104], Andrew Marshall[119,120,121], Emma Matthews[122,123], Mark I McCarthy[33,35,124], Tara Renton[91], Andrew S C Rice[125,126], Tom Vale[10], Suellen M Walker[9,127], Christopher Geoffrey Woods[24,28], David L Bennett[10]; **Primary Immune Disorders (PID)** James E Thaventhiran[128], Hana Lango Allen[3,4], Sinisa Savic[129,130], Hana Alachkar[118], Richard Antrobus[131], Helen E Baxendale[22,23,100,132], Michael J Browning[133], Matthew S Buckland[134], Nichola Cooper[135], J David M Edgar[136,137], William Egner[138], Kimberly C Gilmour[9,127], Sarah Goddard[139], Pavels

Gordins[140], Sofia Grigoriadou[141], Scott Hackett[142], Rosie Hague[143], Grant Hayman[144], Archana Herwadkar[118], Aarnoud P Huissoon[142], Stephen Jolles[145], Peter Kelleher[146,147], Dinakantha Kumararatne[148], Hilary Longhurst[141], Lorena E Lorenzo[141], Paul A Lyons[22], Jesmeen Maimaris[127], Sadia Noorani[149], Alex Richter[131], Crina Samarghitean[3,4], Ravishankar B Sargur[138], W A Carrock Sewell[150], Ilenia Simeoni[3,4], Emily Staples[22], David Thomas[22], Moira J Thomas[151,152], Austen Worth[9], Patrick F K Yong[153], Taco W Kuijpers[154,155], Adrian J Thrasher[127], Kenneth G C Smith[22]; **Primary Membranoproliferative Glomerulonephritis (PMG)** Adam P Levine[11], Omid Sadeghi-Alavijeh[11], Edwin K S Wong[16,156], H Terence Cook[157], Melanie M Y Chan[11], Matthew Hall[158], Claire Harris[156], Paul McAlinden[156], Kevin J Marchbank[156,159], Stephen Marks[9], Heather Maxwell[143], Monika Mozere[11], Julie Wessels[139], Sally A Johnson[156,160], Daniel P Gale[11]; **Pulmonary Arterial Hypertension (PAH)** Marta Bleda[22], Charaka Hadinnapola[22], Matthias Haimel[3,4,22], Emilia Swietlik[22], Harm Bogaard[161], Colin Church[162], Gerry Coghlan[134], Robin Condliffe[163], Paul Corris[156,164], Cesare Danesino[165], Mélanie Eyries[166], Henning Gall[167], Hossein-Ardeschir Ghofrani[135,167], J Simon R Gibbs[89], Barbara Girerd[168,169,170], Simon Holden[84], Arjan Houweling[161], Luke S Howard[89,171], Marc Humbert[168,169,170], David G Kiely[163], Gabor Kovacs[172,173], Allan Lawrie[174], Robert V MacKenzie Ross[175], Jennifer Martin[4,22,43], Shahin Moledina[9], David Montani[168,169,170], Michael Newnham[22,23], Andrea Olschewski[172], Horst Olschewski[172,173], Andrew Peacock[162], Joanna Pepke-Zaba[23], Laura Scelsi[176], Werner Seeger[167], Florent Soubrier[166], Jay Suntharalingam[175], Mark Toshner[22,23], Carmen Treacy[22,23], Richard Trembath[26], Anton Vonk Noordegraaf[161], Quinten Waisfisz[177], John Wharton[135], Martin R Wilkins[135], Stephen J Wort[90,178], Katherine Yates[3,4,22], Stefan Gräf[3,4,22], Nicholas W Morrell[4,22]; **Stem cell and Myeloid Disorders (SMD)** Eleni Louka[31,33], Noemi B Roy[31,32,33], Anupama Rao[9], Philip Ancliff[9], Christian Babbs[31,33], D Mark Layton[51,52], Adam J Mead[31], Jennifer O'Sullivan[75], Steven Okoli[31,33], Irene Roberts[31,32,33]; **Steroid Resistant Nephrotic Syndrome (SRNS)** Moin Saleem[179,180], Agnieszka Bierzynska[179], Carmen Bugarin Diz[26], Elizabeth Colby[179], Melanie N Ekani[20], Simon Satchell[179,181], Ania Koziell[26,27]

Author addresses and affiliations

[1]Department of Clinical Neurosciences, School of Clinical Medicine, University of Cambridge, Cambridge Biomedical Campus, Cambridge, UK. [2]Medical Research Council Mitochondrial Biology Unit, Cambridge Biomedical Campus, Cambridge, UK. [3]Department of Haematology, University of Cambridge, Cambridge Biomedical Campus, Cambridge, UK. [4]NIHR BioResource, Cambridge University Hospitals NHS Foundation, Cambridge Biomedical Campus, Cambridge, UK. [5]Genomics England, Charterhouse Square, London, UK. [6]MRC Clinical Sciences Centre, Faculty of Medicine, Imperial College London, London, UK. [7]Institute of Genetics and Molecular Medicine, University of Edinburgh, Edinburgh, UK. [8]Genetics and Genomic Medicine Programme, UCL Great Ormond Street Institute of Child Health, London, UK. [9]Great Ormond Street Hospital for Children NHS Foundation Trust, London, UK. [10]The Nuffield Department of Clinical Neurosciences, University of Oxford, John Radcliffe Hospital, Oxford, UK. [11]UCL Centre for Nephrology, University College London, London, UK. [12]University College London, London, UK. [13]Evolution and Genomic Sciences, Faculty of Biology, Medicine and Health, University of Manchester, Manchester, UK. [14]Manchester Centre for Genomic Medicine, St Mary's Hospital, Manchester Universities Foundation NHS Trust, Manchester, UK. [15]Newcastle University, Newcastle upon Tyne, UK. [16]Newcastle upon Tyne Hospitals NHS Foundation Trust, Newcastle upon Tyne, UK. [17]Northern Genetics Service, Newcastle upon Tyne Hospitals NHS Foundation Trust, Newcastle upon Tyne, UK. [18]UCL Institute of Cardiovascular Science, University College London, London, UK. [19]Barts Heart Centre, St Bartholomew's Hospital, Barts Health NHS Trust, London, UK. [20]Guy's and St Thomas' Hospital, Guy's and St Thomas' NHS Foundation Trust, London, UK. [21]Clinical Genetics Department, Guy's and St Thomas NHS Foundation Trust, London, UK. [22]Department of Medicine, School of Clinical Medicine, University of Cambridge,

Cambridge Biomedical Campus, Cambridge, UK. [23]Royal Papworth Hospital NHS Foundation Trust, Cambridge, UK. [24]Addenbrookes Hospital, Cambridge University Hospitals NHS Foundation Trust, Cambridge, UK. [25]Department of Molecular Neuroscience, UCL Institute of Neurology, London, UK. [26]King's College London, London, UK. [27]Department of Paediatric Nephrology, Evelina London Children's Hospital, Guy's & St Thomas' NHS Foundation Trust, London, UK. [28]Department of Medical Genetics, Cambridge Institute for Medical Research, University of Cambridge, Cambridge Biomedical Campus, Cambridge, UK. [29]NIHR Cambridge Biomedical Research Centre, Cambridge Biomedical Campus, Cambridge, UK. [30]Stroke Research Group, Department of Clinical Neurosciences, University of Cambridge, Cambridge Biomedical Campus, Cambridge, UK. [31]MRC Molecular Haematology Unit, MRC Weatherall Institute of Molecular Medicine, University of Oxford, Oxford, UK. [32]Department of Paediatrics, Weatherall Institute of Molecular Medicine, University of Oxford, Oxford, UK. [33]NIHR Oxford Biomedical Research Centre, Oxford University Hospitals Trust, Oxford, UK. [34]Institute of Genetic Medicine, Newcastle University, Newcastle upon Tyne, UK. [35]Wellcome Centre for Human Genetics, University of Oxford, Oxford, UK. [36]Department of Cardiovascular Medicine, Radcliffe Department of Medicine, University of Oxford, Oxford, UK. [37]Oxford University Hospitals NHS Foundation Trust, Oxford, UK. [38]Moorfields Eye Hospital NHS Foundation Trust, London, UK. [39]UCL Institute of Ophthalmology, University College London, London, UK. [40]MRC Weatherall Institute of Molecular Medicine, University of Oxford, John Radcliffe Hospital, Oxford, UK. [41]Division of Women's Health, King's College London, London, UK. [42]Institute of Reproductive and Developmental Biology, Surgery and Cancer, Hammersmith Hospital, Imperial College Healthcare NHS Trust, London, UK. [43]Department of Public Health and Primary Care, University of Cambridge, Cambridge, UK. [44]Wellcome Sanger Institute, Wellcome Genome Campus, Hinxton, Cambridge, UK. [45]NHS Blood and Transplant, Cambridge Biomedical Campus, Cambridge, UK. [46]British Heart Foundation Cambridge Centre of Excellence, University of Cambridge, Cambridge, UK. [47]Department of Renal Medicine, Addenbrookes Hospital, Cambridge University Hospitals NHS Foundation Trust, Cambridge, UK. [48]William Harvey Research Institute, NIHR Biomedical Research Centre at Barts, Queen Mary University of London, London, UK. [49]MRC Biostatistics Unit, Cambridge Institute of Public Health, University of Cambridge, Cambridge, UK. [50]The Katharine Dormandy Haemophilia Centre and Thrombosis Unit, Royal Free London NHS Foundation Trust, London, UK. [51]Department of Haematology, Hammersmith Hospital, Imperial College Healthcare NHS Trust, London, UK. [52]Centre for Haematology, Imperial College London, London, UK. [53]The Royal London Hospital, Barts Health NHS Foundation Trust, London, UK. [54]Department of Haematology, Cambridge University Hospitals NHS Foundation Trust, Cambridge, UK. [55]Queen Mary University of London, London, UK. [56]School of Cellular and Molecular Medicine, University of Bristol, Bristol, UK. [57]University Hospitals Bristol NHS Foundation Trust, Bristol, UK. [58]Queens Centre for Haematology and Oncology, Castle Hill Hospital, Hull and East Yorkshire NHS Trust, Cottingham, UK. [59]Hull York Medical School, University of Hull, Hull, UK. [60]Center for Clinical Transfusion Medicine, University Hospital of Tübingen, Tübingen, Germany. [61]Haematology Department, Royal Victoria Infirmary, The Newcastle upon Tyne Hospitals NHS Foundation Trust, Newcastle upon Tyne, UK. [62]Southampton General Hospital, University Hospital Southampton NHS Foundation Trust, Southampton, UK. [63]Institute of Infection and Immunity, School of Medicine Cardiff University, Cardiff, UK. [64]Oxford Haemophilia and Thrombosis Centre, Oxford University Hospitals NHS Trust, Oxford Comprehensive Biomedical Research Centre, Oxford, UK. [65]The Roald Dahl Haemostasis and Thrombosis Centre, The Royal Liverpool University Hospital, Liverpool, UK. [66]Medical School and School of Biomedical Sciences, Faculty of Health and Medical Sciences, The University of Western Australia, Crawley, Australia. [67]Haemophilia Centre, Kent & Canterbury Hospital, East Kent Hospitals University Foundation Trust, Canterbury, UK. [68]Salisbury District Hospital, Salisbury NHS Foundation Trust, Salisbury, UK. [69]Haemophilia, Haemostasis and Thrombosis

Centre, Hampshire Hospitals NHS Foundation Trust, Basingstoke, UK. [70]INSERM UMR 1170, Gustave Roussy Cancer Campus, Universite Paris-Saclay, Villejuif, France. [71]Service d'Hematologie biologique, Centre de Reference des Pathologies Plaquettaires, Hopital Armand Trousseau, Assistance Publique-Hopitaux de Paris, Paris, France. [72]Section of Internal and Cardiovascular Medicine, University of Perugia, Perugia, Italy. [73]Division of Hematology, The Children's Hospital of Philadelphia, Philadelphia, USA. [74]Department of Pediatrics, Perelman School of Medicine at the University of Pennsylvania, Philadelphia, USA. [75]Department of Haematology, Guy's and St Thomas' NHS Foundation Trust, London, UK. [76]Department of Haematology, Great Ormond Street Hospital for Children NHS Foundation Trust, London, UK. [77]The Arthur Bloom Haemophilia Centre, University Hospital of Wales, Cardiff, UK. [78]Department of Cardiovascular Sciences, Center for Molecular and Vascular Biology, KULeuven, Leuven, Belgium. [79]Beth Israel Deaconess Medical Centre and Harvard Medical School, Boston, USA. [80]University College London Hospitals NHS Foundation Trust, London, UK. [81]Oxford Haemophilia and Thrombosis Centre, The Churchill Hospital, Oxford University Hospitals NHS Trust, Oxford, UK. [82]Glasgow Royal Infirmary, NHS Greater Glasgow and Clyde, Glasgow, UK. [83]Department of Neurology, Sheffield Teaching Hospitals NHS Foundation Trust, Sheffield, UK. [84]Department of Clinical Genetics, Addenbrookes Hospital, Cambridge University Hospitals NHS Foundation Trust, Cambridge, UK. [85]Institute of Neuroscience and Psychology, University of Glasgow, Glasgow, UK. [86]Department of Neurology, Leeds Teaching Hospital NHS Trust, Leeds, UK. [87]North East Thames Regional Genetics Service, Great Ormond Street Hospital for Children NHS Foundation Trust, London, UK. [88]Division of Medical Genetics, IWK Health Centre, Dalhousie University, Halifax, Canada. [89]National Heart and Lung Institute, Imperial College London, London, UK. [90]Royal Brompton Hospital, Royal Brompton and Harefield NHS Foundation Trust, London, UK. [91]King's College Hospital NHS Foundation Trust, London, UK. [92]MRC London Institute of Medical Sciences, Imperial College London, London, UK. [93]National Heart Research Institute Singapore, National Heart Centre Singapore, Singapore, Singapore. [94]Division of Cardiovascular & Metabolic Disorders, Duke-National University of Singapore, Singapore, Singapore. [95]Department of Biotechnology, Graduate School of Engineering, Osaka University, Suita, Osaka, Japan. [96]Women's Health Research Centre, Surgery and Cancer, Faculty of Medicine, Hammersmith Hospital, Imperial College Healthcare NHS Trust, London, UK. [97]Ramón Sardá Mother's and Children's Hospital, Buenos Aires, Argentina. [98]Robinson Research Institute, Discipline of Obstetrics and Gynaecology, The University of Adelaide, Women's and Children's Hospital, Adelaide, Australia. [99]Department of Molecular and Clinical Medicine, Sahlgrenska Academy, University of Gothenburg, Gothenburg, Sweden. [100]Department of Paediatrics, School of Clinical Medicine, University of Cambridge, Cambridge Biomedical Campus, Cambridge, UK. [101]Ophthalmology Department, UCSF School of Medicine, San Francisco, USA. [102]Wellcome Centre for Mitochondrial Research, Institute of Genetic Medicine, Newcastle University, Newcastle upon Tyne, UK. [103]Institute of Genetic Medicine, Newcastle University, Newcastle upon Tyne, UK. [104]John Walton Muscular Dystrophy Research Centre, Institute of Genetic Medicine, Newcastle University, Newcastle upon Tyne, UK. [105]Cancer Research UK Cambridge Centre, Cambridge Biomedical Campus, Cambridge, UK. [106]Yorkshire Regional Genetics Service, Chapel Allerton Hospital, Leeds Teaching Hospitals NHS Trust, Leeds, UK. [107]Department of Clinical Genetics, Royal Devon & Exeter Hospital, Royal Devon and Exeter NHS Foundation Trust, Exeter, UK. [108]West Midlands Regional Genetics Service, Birmingham Women's and Children's NHS Foundation Trust, Birmingham, UK. [109]Manchester University NHS Foundation Trust, Manchester, UK. [110]Department of Clinical Genetics, Liverpool Women's NHS Foundation, Liverpool, UK. [111]Department of Clinical Genetics, St George's University Hospitals NHS Foundation Trust, London, UK. [112]Department of Clinical Genetics, Guy's and St Thomas' NHS Foundation Trust, London, UK. [113]East Anglian Medical Genetics Service, Cambridge University Hospitals NHS Foundation Trust, Cambridge, UK. [114]Department of Clinical Genetics, Nottingham University

Hospitals NHS Trust, Nottingham, UK. [115]Department of Medical Genetics and NIHR Cambridge Biomedical Research Centre, University of Cambridge, Cambridge, UK. [116]Developmental Neurosciences, UCL Great Ormond Street Institute of Child Health, London, UK. [117]Department of Neurology, Great Ormond Street Hospital for Children NHS Foundation Trust, London, UK. [118]Salford Royal NHS Foundation Trust, Salford, UK. [119]Faculty of Medical and Human Sciences, Centre for Endocrinology and Diabetes, Institute of Human Development, University of Manchester, Manchester, UK. [120]Department of Clinical Neurophysiology, Manchester Royal Infirmary, Central Manchester University Hospitals National Health Service Foundation Trust, Manchester Academic Health Science Centre, Manchester, UK. [121]National Institute for Health Research/Wellcome Trust Clinical Research Facility, Manchester, UK. [122]The National Hospital for Neurology and Neurosurgery, University College London Hospitals NHS Foundation Trust, London, UK. [123]MRC Centre for Neuromuscular Diseases, Department of Molecular Neuroscience, UCL Institute of Neurology, London, UK. [124]Oxford Centre for Diabetes, Endocrinology and Metabolism, University of Oxford, Churchill Hospital, Oxford, UK. [125]Pain Research, Department of Surgery and Cancer, Faculty of Medicine, Imperial College London, London, UK. [126]Pain Medicine, Chelsea and Westminster Hospital NHS Foundation Trust, London, UK. [127]UCL Great Ormond Street Institute of Child Health, London, UK. [128]MRC Toxicology Unit, School of Biological Sciences, University of Cambridge, Cambridge, UK. [129]The Department of Clinical Immunology and Allergy and The NIHR Leeds Biomedical Research Centre, Leeds, UK. [130]Leeds Institute of Rheumatic and Musculoskeletal Medicine, St James's University Hospital, Leeds, UK. [131]University Hospitals Birmingham NHS Foundation Trust, Birmingham, UK. [132]Division of Clinical Biochemistry and Immunology, Cambridge University Hospitals NHS Foundation Trust, Cambridge, UK. [133]Department of Immunology, Leicester Royal Infirmary, Leicester, UK. [134]Royal Free London NHS Foundation Trust, London, UK. [135]Department of Medicine, Imperial College London, London, UK. [136]Regional Immunology Service, The Royal Hospitals, Belfast, UK. [137]Queen's University Belfast, Belfast, UK. [138]Sheffield Teaching Hospitals NHS Foundation Trust, Sheffield, UK. [139]University Hospitals of North Midlands NHS Trust, Stoke-on-Trent, UK. [140]East Yorkshire Regional Adult Immunology and Allergy Unit, Hull Royal Infirmary, Hull & East Yorkshire Hospitals NHS Trust, Hull, UK. [141]Barts Health NHS Foundation Trust, London, UK. [142]Birmingham Heartlands Hospital, University Hospitals Birmingham NHS Foundation Trust, Birmingham, UK. [143]Royal Hospital for Children, NHS Greater Glasgow and Clyde, Glasgow, UK. [144]Epsom & St Helier University Hospitals NHS Trust, London, UK. [145]Immunodeficiency Centre for Wales, University Hospital of Wales, Cardiff, IUK. [146]Centre for Immunology & Vaccinology , Chelsea & Westminster Hospital , Department of Medicine, Imperial College London, London, UK. [147]Department of Respiratory Medicine Royal Brompton & Harefield NHS Foundation Trust, London, UK. [148]Department of Clinical Immunology, Addenbrookes Hospital, Cambridge University Hospitals NHS Foundation Trust, Cambridge, UK. [149]Sandwell and West Birmingham Hospitals NHS Trust, Birmingham, UK. [150]Scunthorpe General Hospital, Northern Lincolnshire and Goole NHS Foundation Trust, Scunthorpe, UK. [151]Gartnavel General Hospital, NHS Greater Glasgow and Clyde, Glasgow, UK. [152]Queen Elizabeth University Hospital, Glasgow, UK. [153]Frimley Park Hospital, NHS Frimley Health Foundation Trust, Camberley, UK. [154]Department of Pediatric Hematology, Immunology, Rheumatology and Infectious Diseases, Emma Children's Hospital, Academic Medical Center (AMC), University of Amsterdam, Amsterdam, The Netherlands. [155]Department of Blood Cell Research, Sanquin, Amsterdam, The Netherlands. [156]Institute of Cellular Medicine, Faculty of Medical Sciences, Newcastle University, Newcastle upon Tyne, UK. [157]Imperial College Renal and Transplant Centre, Hammersmith Hospital, Imperial College Healthcare NHS Trust, London, UK. [158]Nottingham University Hospitals NHS Trust, Nottingham, UK. [159]The National Renal Complement Therapeutics Centre, Royal Victoria Infirmary, Newcastle upon Tyne, UK. [160]Department of Paediatric Nephrology, Great North Children's Hospital, Newcastle upon Tyne Hospitals NHS

Foundation Trust, Newcastle upon Tyne, UK. [161]Department of Pulmonary Medicine, VU University Medical Centre, Amsterdam, The Netherlands. [162]Golden Jubilee National Hospital, Glasgow, UK. [163]Sheffield Pulmonary Vascular Disease Unit, Royal Hallamshire Hospital NHS Foundation Trust, Sheffield, UK. [164]National Pulmonary Hypertension Service (Newcastle), The Newcastle upon Tyne Hospitals NHS Foundation Trust, Newcastle upon Tyne, UK. [165]Department of Molecular Medicine, General Biology, and Medical Genetics Unit, University of Pavia, Pavia, Italy. [166]Departement de Genetique & ICAN, Hopital Pitie-Salpetriere, Assistance Publique Hopitaux de Paris, Paris, France. [167]University of Giessen and Marburg Lung Center (UGMLC), Giessen, Germany. [168]Univ. Paris-Sud, Faculty of Medicine, University Paris-Saclay, Le Kremlin Bicetre, France. [169]Service de Pneumologie, Centre de Reference de l'Hypertension Pulmonaire, Hopital Bicetre (Assistance Publique Hopitaux de Paris), Le Kremlin Bicetre, France. [170]INSERM U999, Hospital Marie Lannelongue, Le Plessis Robinson, France. [171]National Pulmonary Hypertension Service, Imperial College Healthcare NHS Trust, London, UK. [172]Ludwig Boltzmann Institute for Lung Vascular Research, Graz, Austria. [173]Dept of Internal Medicine, Division of Pulmonology, Medical University of Graz, Graz, Austria. [174]Department of Infection, Immunity & Cardiovascular Disease, University of Sheffield, Sheffield, UK. [175]Royal United Hospitals Bath NHS Foundation Trust, Bath, UK. [176]Division of Cardiology, Fondazione IRCCS Policlinico S. Matteo, Pavia, Italy. [177]Department of Clinical Genetics, VU University Medical Centre, Amsterdam, The Netherlands. [178]Imperial College London, London, UK. [179]Bristol Renal and Children's Renal Unit, Bristol Medical School, University of Bristol, Bristol, UK. [180]Bristol Royal Hospital for Children, University Hospitals Bristol NHS Foundation Trust, Bristol, UK. [181]North Bristol NHS Trust, Bristol, UK.

Supplementary Figures

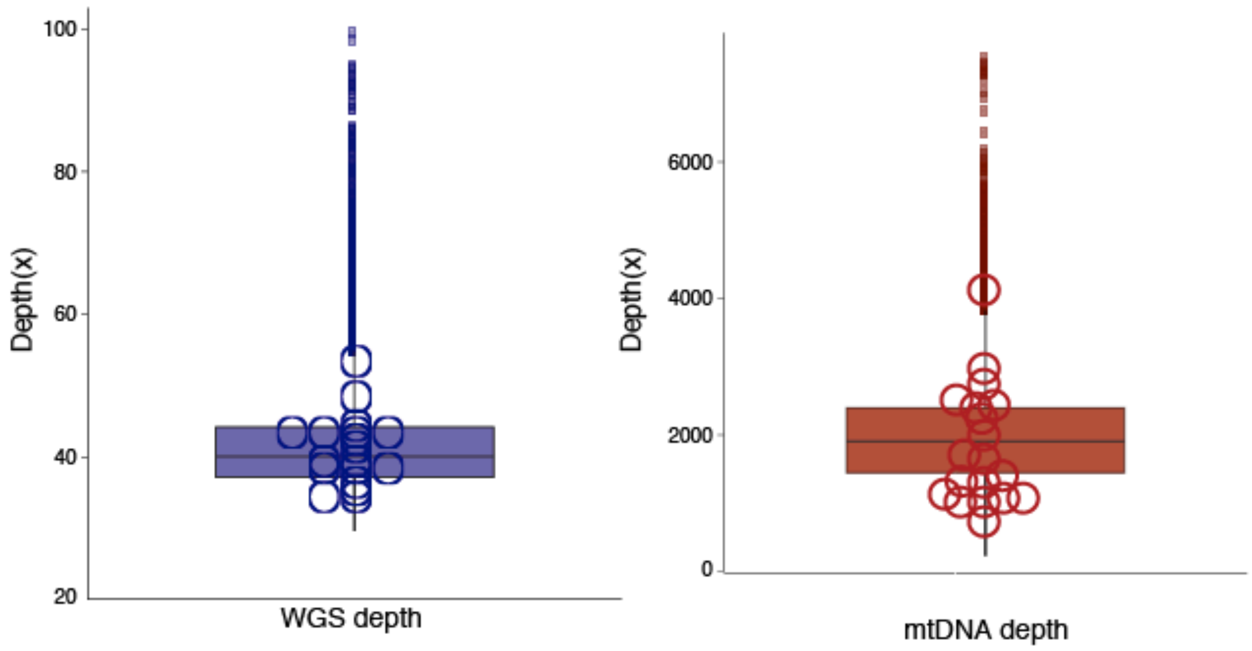

Supplementary Figure 1. Box-whisker plots of mean depth of whole genome sequences (WGS) and mitochondrial genome sequences (mtDNA) from 11,035 trios. The middle "box" represents the median, lower and upper quartile of the data. The upper and lower whiskers represent the data outside the middle 50%. The dots represent the data outside 25% - 75% of the data. Circles are the depth of 7 families where father and offspring carrying the mixed haplotypes (**Supplementary Table 5**).

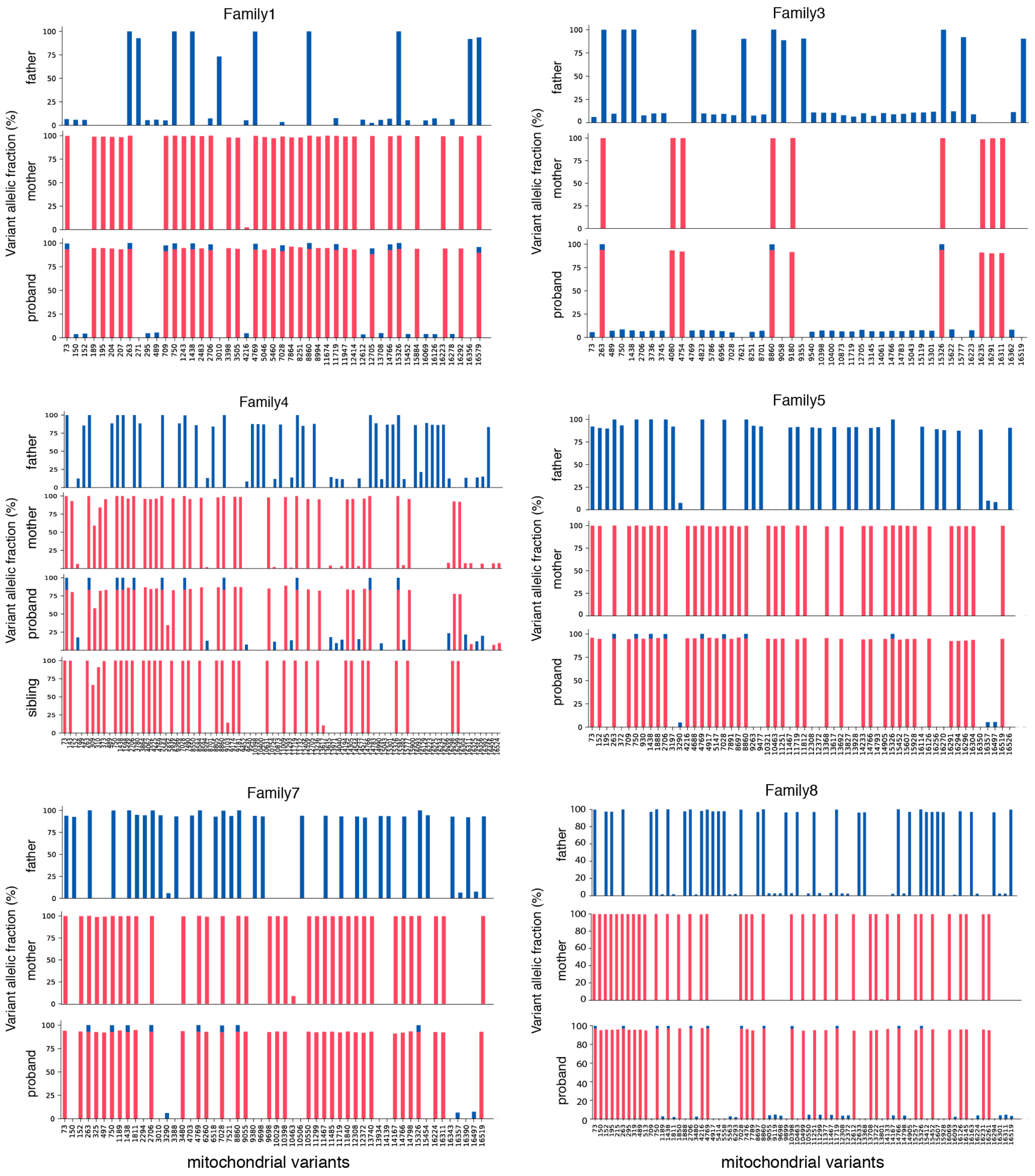

Supplementary Figure 2 MtDNA mixed haplotype patterns observed in 6 families. See Fig. 2a in the main text for details.

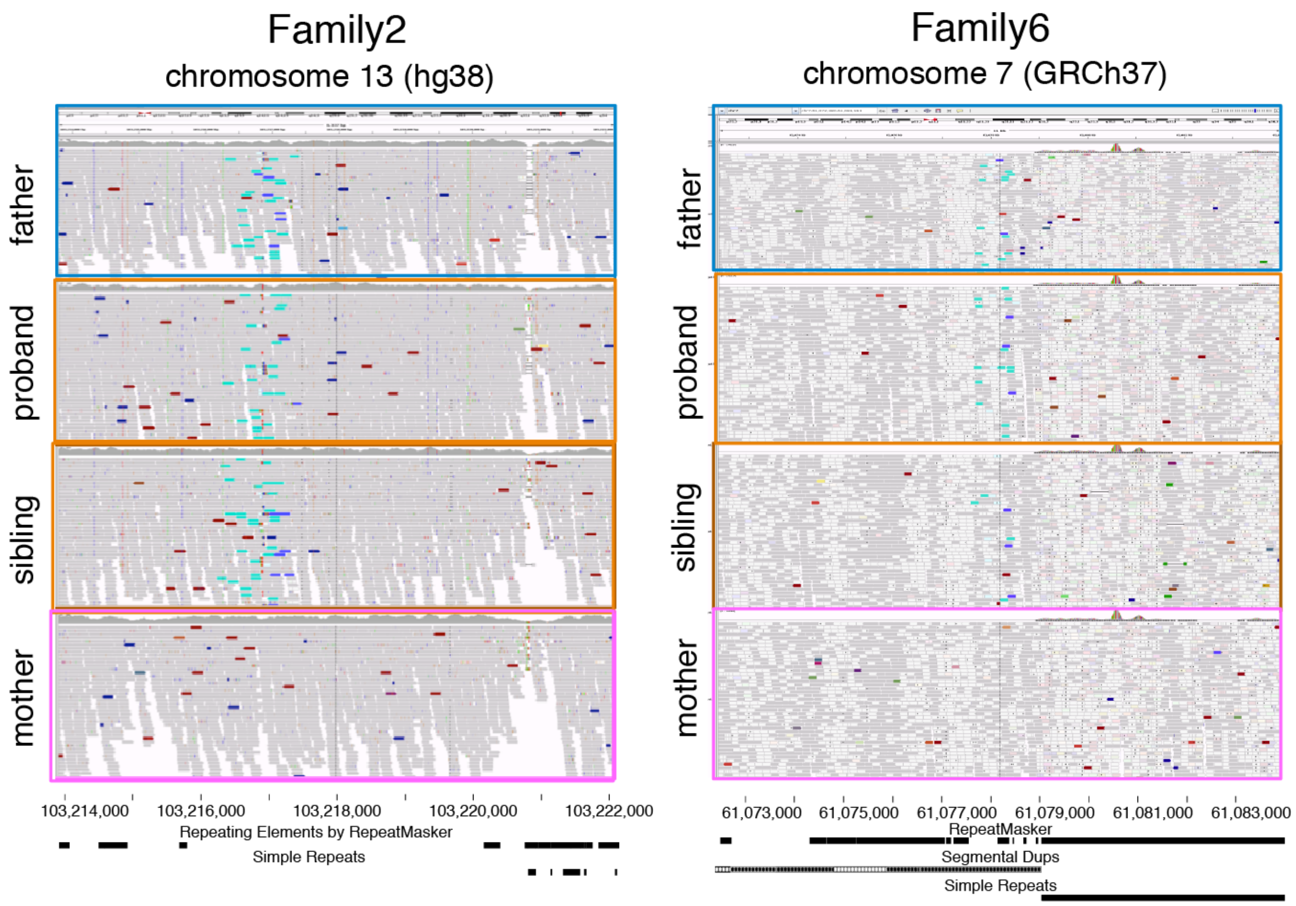

Supplementary Figure 3. IGV screenshots of the aligned reads corresponding to NUMTs on the nuclear DNA in family 2 (left) and family 6 (right). Teal bars indicate the aligned reads which mapped to the nuclear DNA where their mates mapped to the mtDNA. The genome position, repeats and segmental duplications tracks from UCSC genome browser are shown at the bottom.

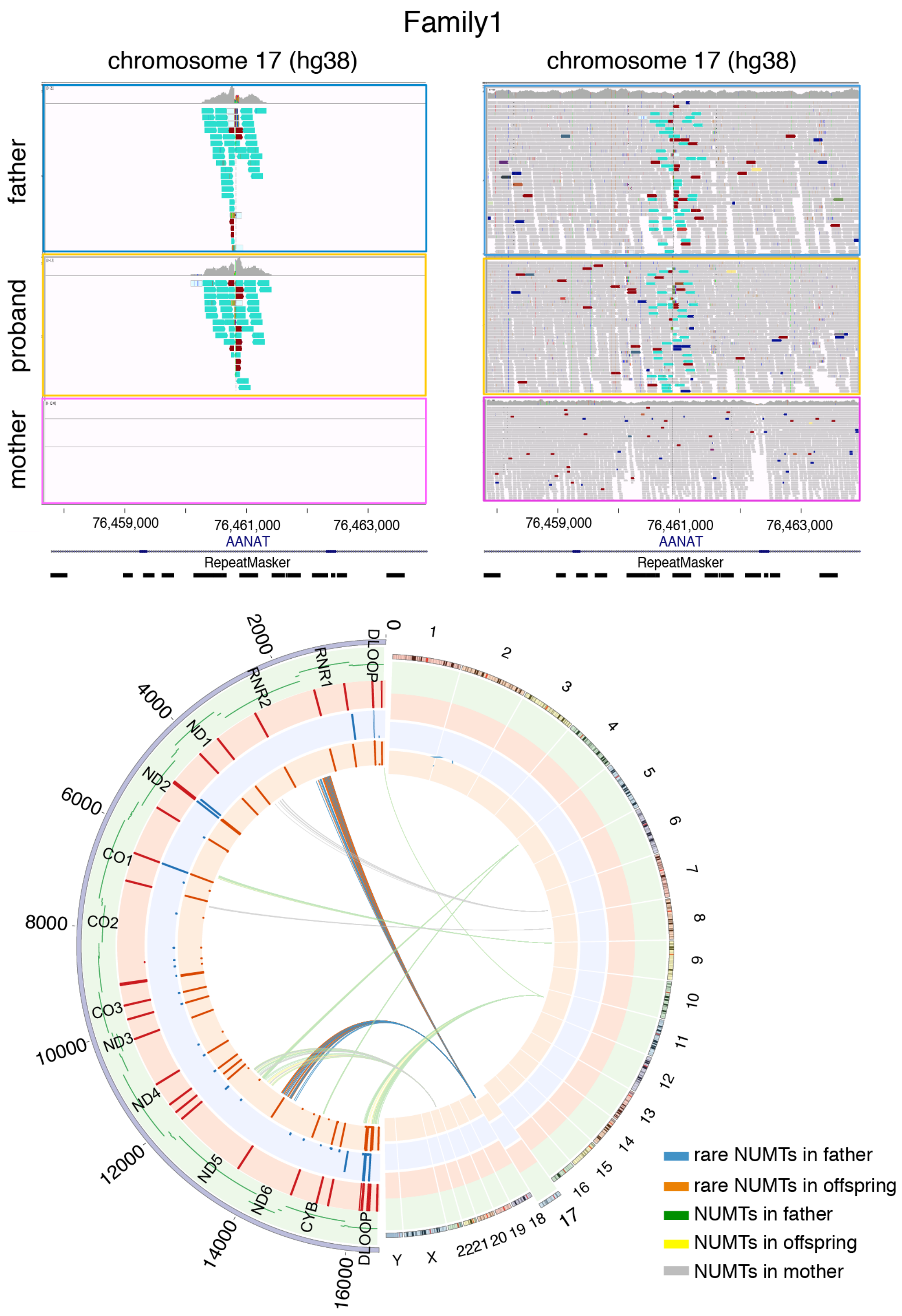

Family1
chromosome 17 (hg38)
chromosome 17 (hg38)
father
proband
mother
76,459,000
76,461,000
76,463,000
AANAT
RepeatMasker
76,459,000
76,461,000
76,463,000
AANAT
RepeatMasker
0
2000
4000
6000
8000
10000
12000
14000
16000
DLOOP
RNR1
RNR2
ND1
ND2
CO1
CO2
CO3
ND3
ND4
ND5
ND6
CYB
DLOOP
1
2
3
4
5
6
7
8
9
10
11
12
13
14
15
16
17
18
19
20
21
22
X
Y
rare NUMTs in father
rare NUMTs in offspring
NUMTs in father
NUMTs in offspring
NUMTs in mother

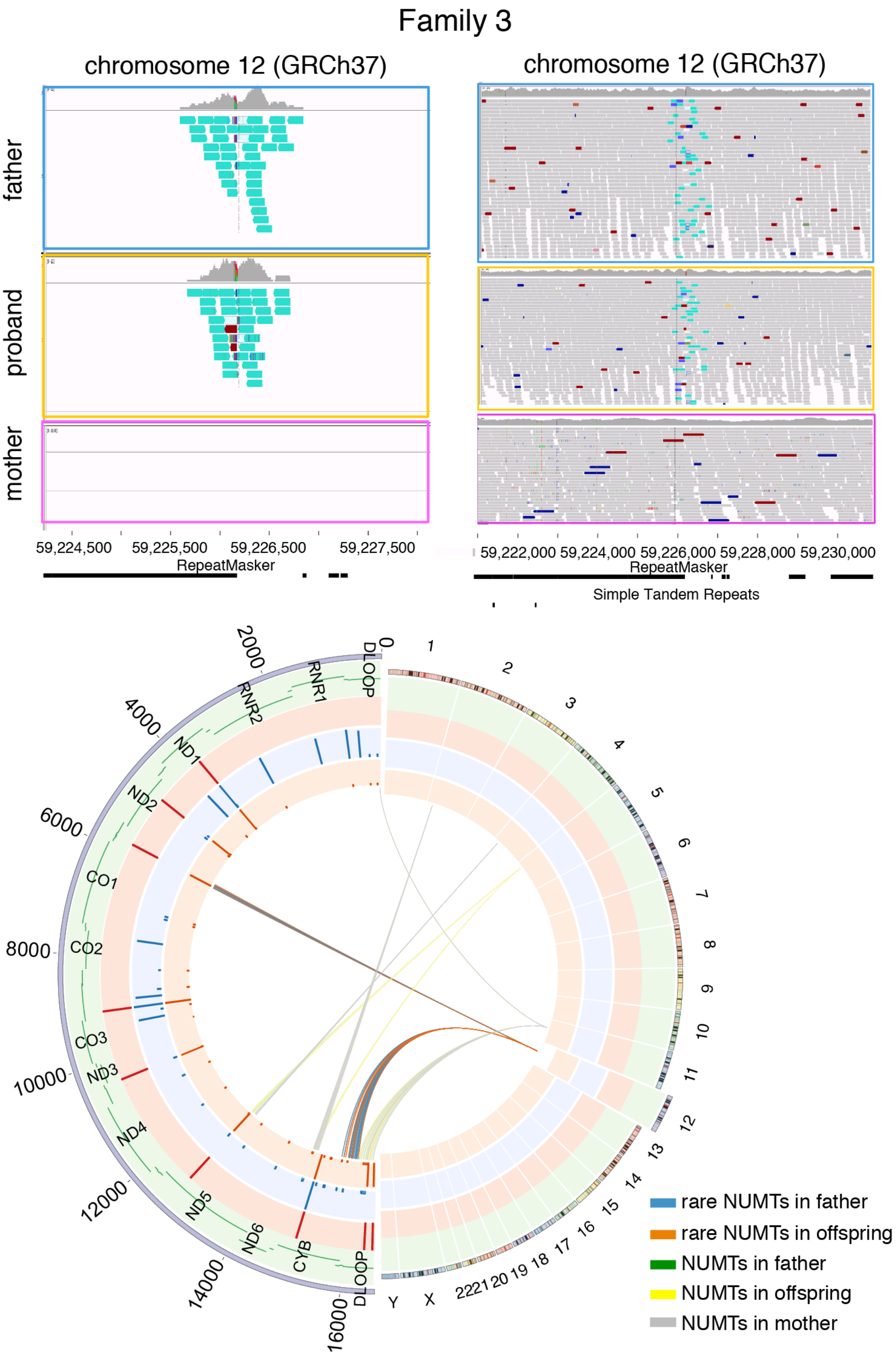
Family 3
chromosome 12 (GRCh37)
chromosome 12 (GRCh37)
father
proband
mother
59,224,500
59,225,500
59,226,500
59,227,500
RepeatMasker
59,222,000 59,224,000 59,226,000 59,228,000 59,230,000
RepeatMasker
Simple Tandem Repeats
DLOOP
RNR1
RNR2
ND1
ND2
CO1
CO2
CO3
ND3
ND4
ND5
ND6
CYB
DLOOP
0
2000
4000
6000
8000
10000
12000
14000
16000
1
2
3
4
5
6
7
8
9
10
11
12
13
14
15
16
17
18
19
20
21
22
X
Y
rare NUMTs in father
rare NUMTs in offspring
NUMTs in father
NUMTs in offspring
NUMTs in mother

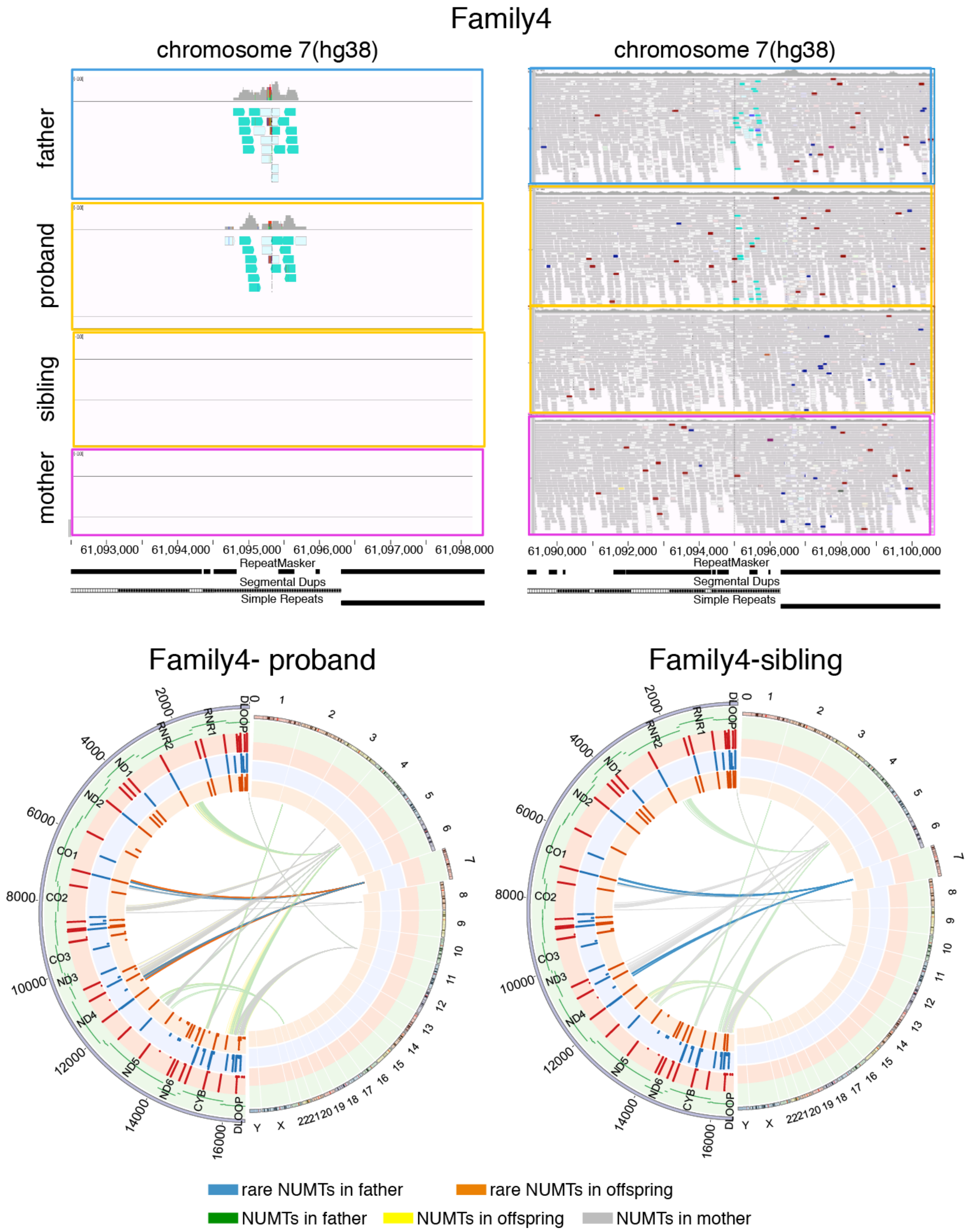
Family4
chromosome 7(hg38)
chromosome 7(hg38)
father
proband
sibling
mother
61,093,000
61,094,000
61,095,000
61,096,000
61,097,000
61,098,000
RepeatMasker
Segmental Dups
Simple Repeats
61,090,000
61,092,000
61,094,000
61,096,000
61,098,000
61,100,000
RepeatMasker
Segmental Dups
Simple Repeats
Family4- proband
Family4-sibling
rare NUMTs in father
rare NUMTs in offspring
NUMTs in father
NUMTs in offspring
NUMTs in mother

Family 5

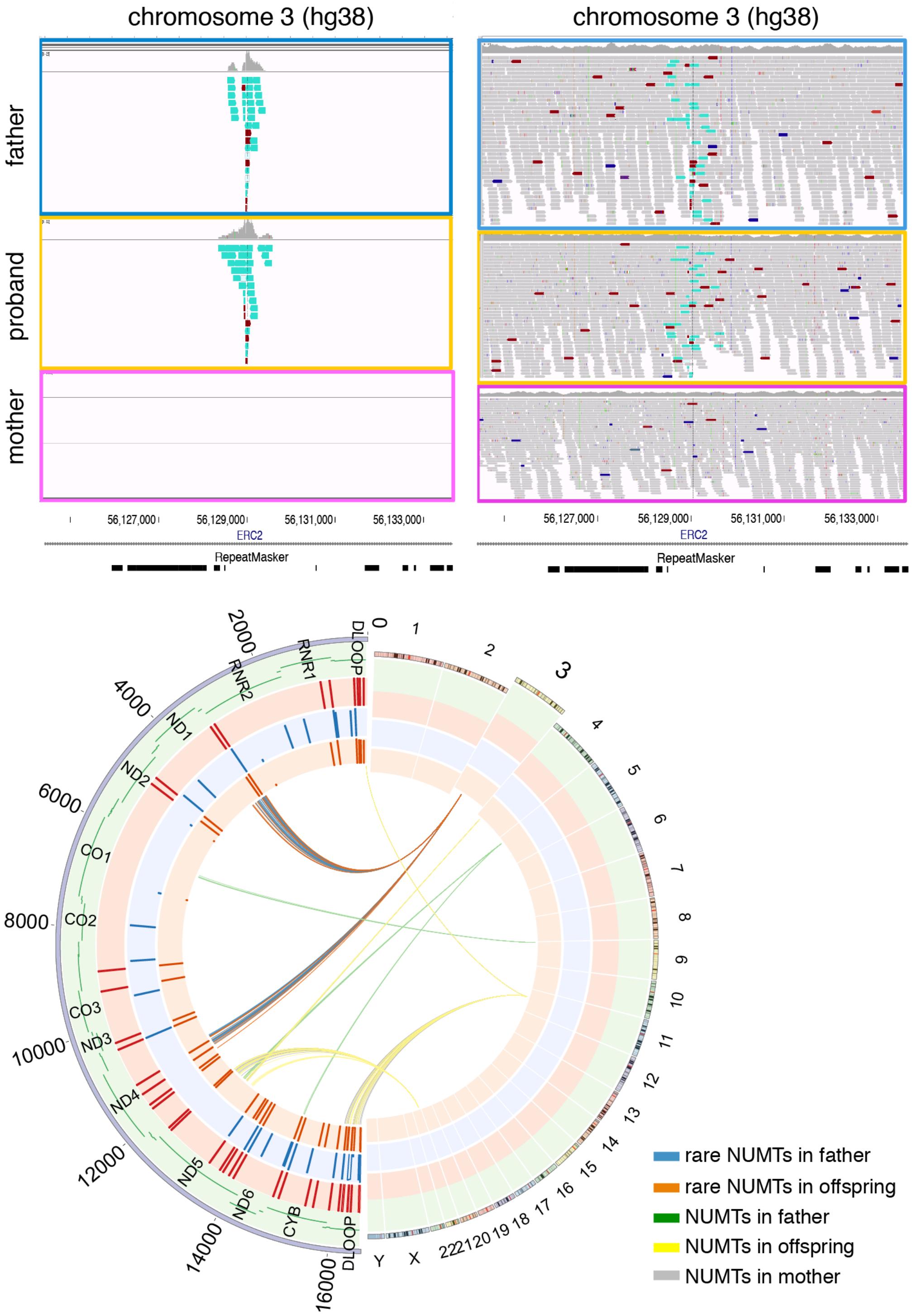
chromosome 3 (hg38)
chromosome 3 (hg38)
father
proband
mother
56,127,000
56,129,000
56,131,000
56,133,000
ERC2
RepeatMasker
rare NUMTs in father
rare NUMTs in offspring
NUMTs in father
NUMTs in offspring
NUMTs in mother

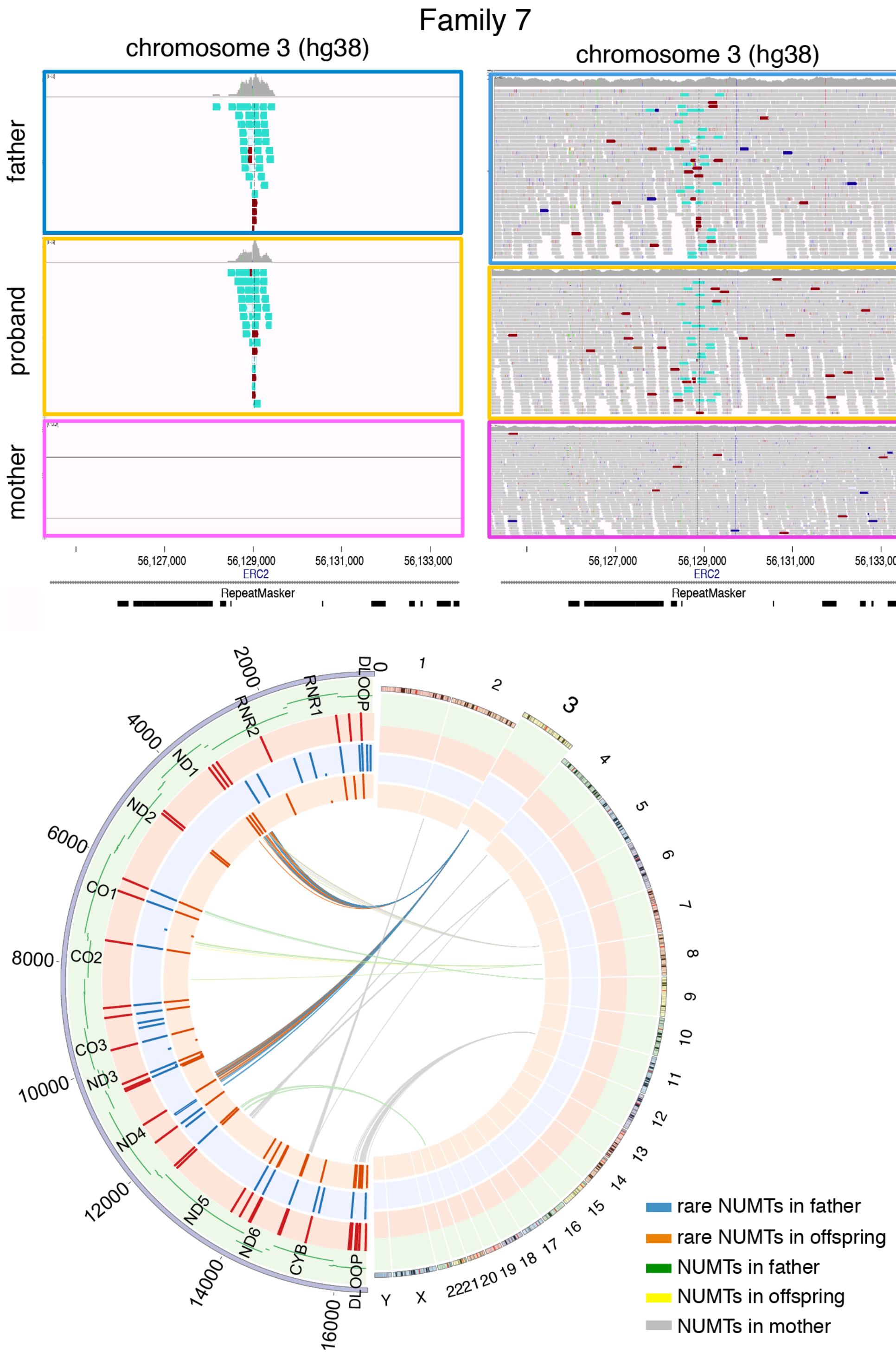

Family 7
chromosome 3 (hg38)
chromosome 3 (hg38)
father
proband
mother
56,127,000
56,129,000
56,131,000
56,133,000
ERC2
RepeatMasker
DLOOP
RNR1
RNR2
ND1
ND2
CO1
CO2
CO3
ND3
ND4
ND5
ND6
CYB
0
2000
4000
6000
8000
10000
12000
14000
16000
1
2
3
4
5
6
7
8
9
10
11
12
13
14
15
16
17
18
19
20
21
22
X
Y
rare NUMTs in father
rare NUMTs in offspring
NUMTs in father
NUMTs in offspring
NUMTs in mother

Supplementary Figure 4 Mapping NUMTs in 5 families. Within each family **(Top)** IGV screenshots show aligned reads corresponding to NUMTs. Top left: The alignment of the discordant and split reads corresponding to NUMTs on the nuclear DNA. Top right: The alignment of all aligned reads corresponding to NUMTs on the nuclear DNA. Teal bars indicate the aligned reads which mapped to the nuclear DNA where their mates mapped to the mtDNA. The genome position, repeats and segmental duplications tracks from UCSC genome browser are shown at the bottom. **(Bottom)** Circos plots show the detected variants and NUMTs. Circles from the outside to the inside indicate the following: (1) position of a variant on the mtDNA; (2) regions corresponding to the different mtDNA genes; (3) variants identified in the mother where the radial axis corresponds to the allele fraction (AF); (4) variants identified in the father; (5) variants identified in the offspring, proband (left) and sibling (right) in family 4 are shown, respectively; (6) NUMTs observed in the family, proband (left) and sibling (right) in family 4 are shown, respectively.

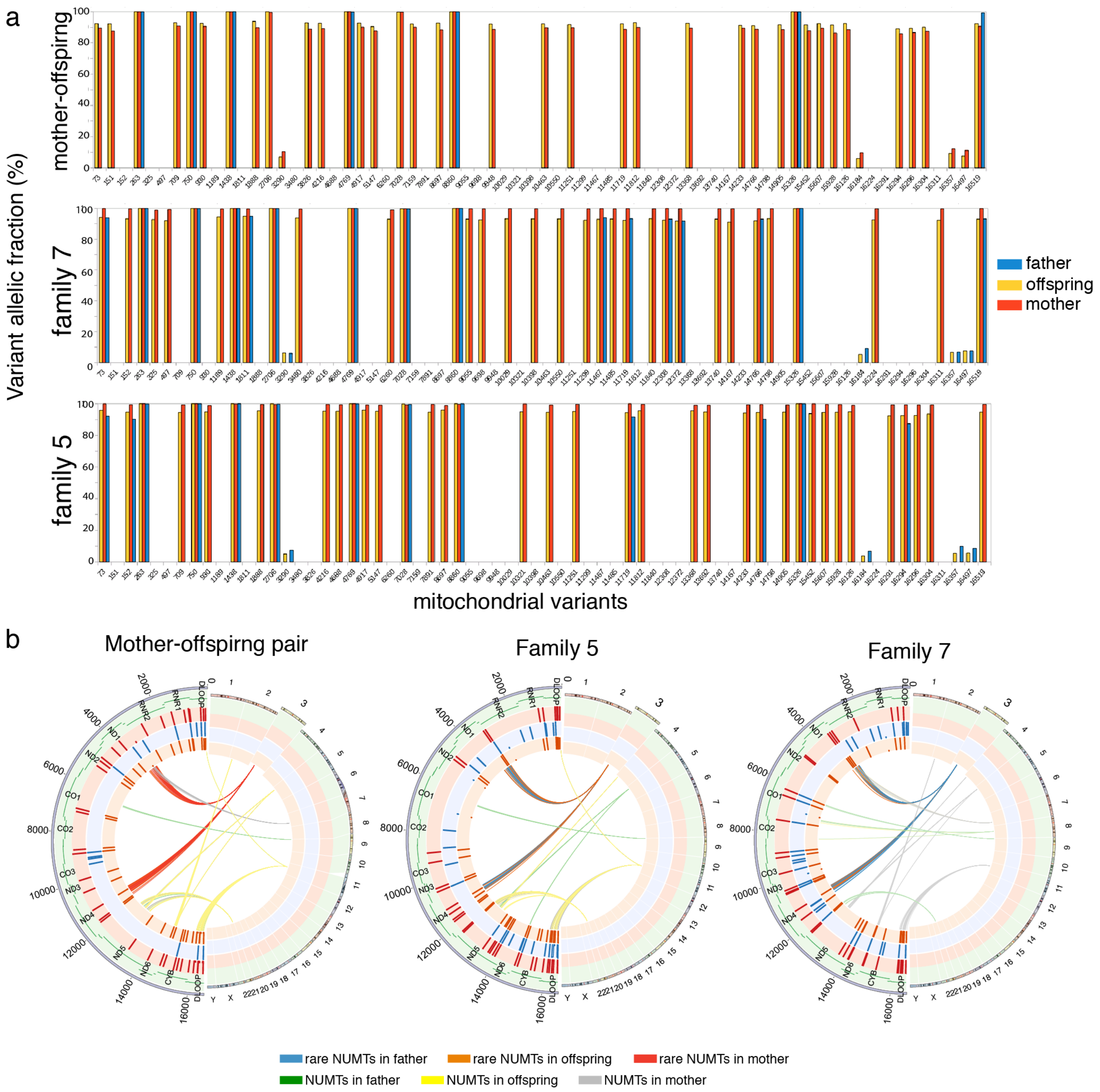
a
b
Variant allelic fraction (%)
mother-offspirng
family 7
family 5
mitochondrial variants
father
offspring
mother
Mother-offspirng pair
Family 5
Family 7
rare NUMTs in father
rare NUMTs in offspring
rare NUMTs in mother
NUMTs in father
NUMTs in offspring
NUMTs in mother

Supplementary Figure 5. Mixed haplotype patterns and circos plots showing the variants and NUMTs observed in family 5, family 7 and one mother-offspring pair. (a) Mixed haplotype patterns observed in family 5, family 7 and one mother-offspring pair. **(b)** Circos plots show the detected variants and NUMTs in family 5, family 7 and one mother-offspring pair. Circles from the outside to the inside indicate the following: (1) position of a variant on the mtDNA; (2) regions corresponding to the different mtDNA genes; (3) variants identified in the mother where the radial axis corresponds to the AF; (4) variants identified in the father; (5) variants identified in the offspring; (6) NUMTs observed in the family.

Transmitted NUMTs in Family 8

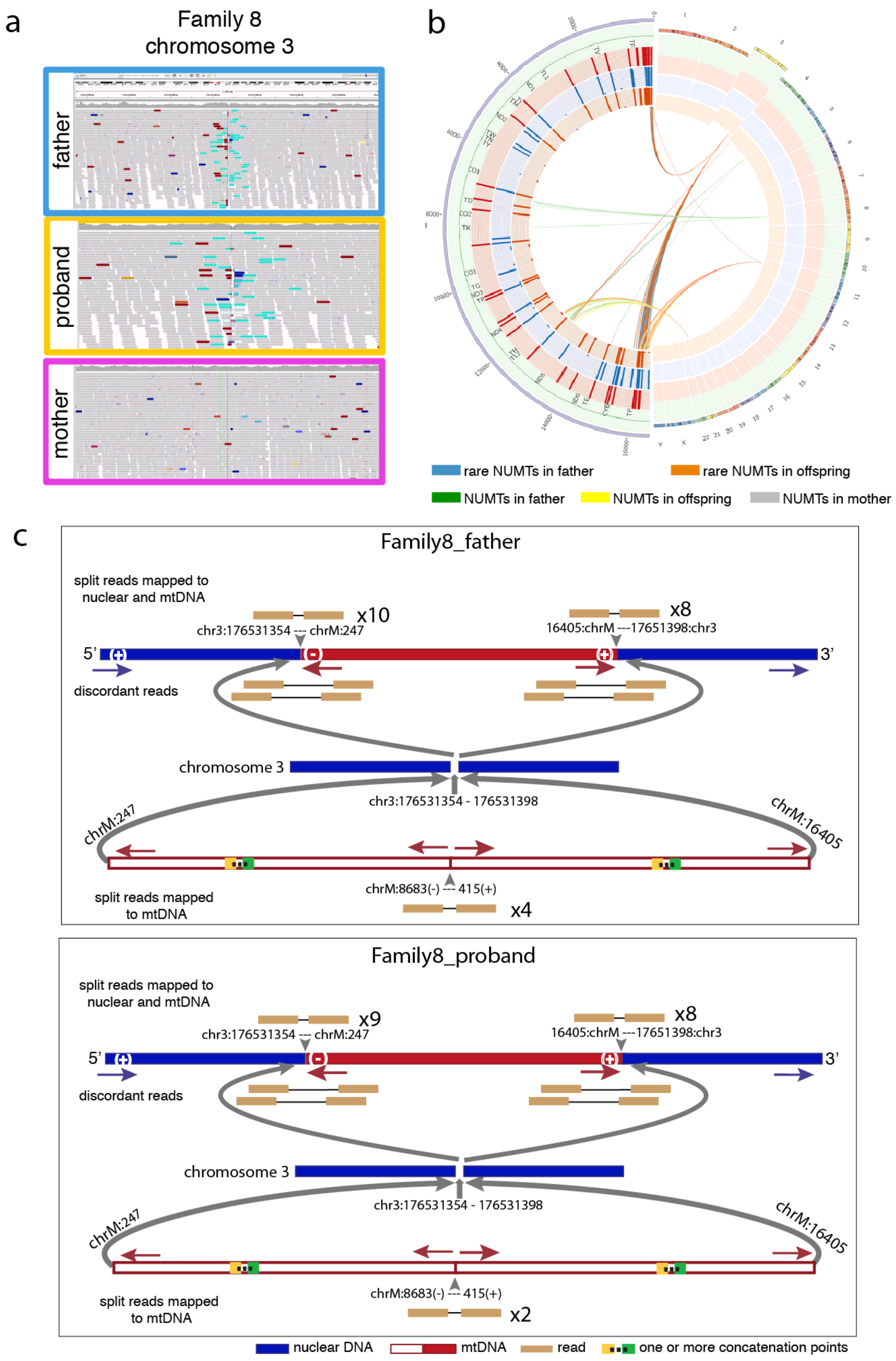

Supplementary Figure 6. Variants and NUMTs observed in family 8 with father and proband carrying the mixed haplotype (haplotype fraction < 5%). **(a)** IGV screenshots show aligned reads corresponding to NUMTs. Teal bars indicate the aligned reads which mapped to the nuclear DNA where their mates mapped to the mtDNA. **(b)** Circos plots shows the detected variants and NUMTs in each family. Circles from the outside to the inside indicate the following: (1) position of a variant on the mtDNA; (2) regions corresponding to the different mtDNA genes; (3) variants identified in the mother where the radial axis corresponds to the AF; (4) variants identified in the father; (5) variants identified in the offspring; (6) NUMTs observed in the family. **(c)** Split reads align to both nuclear DNA and mtDNA-derived sequences (top). Discordant reads are paired reads where one end aligns to mtDNA-derived and the other end aligns to nuclear DNA (middle). Possible constructed concatemer is shown at the bottom with observed supporting split reads. The positions of breakpoints (bp) are shown on both nuclear DNA (top) and mtDNA (bottom).

nonTransmitted NUMTs in Family 9

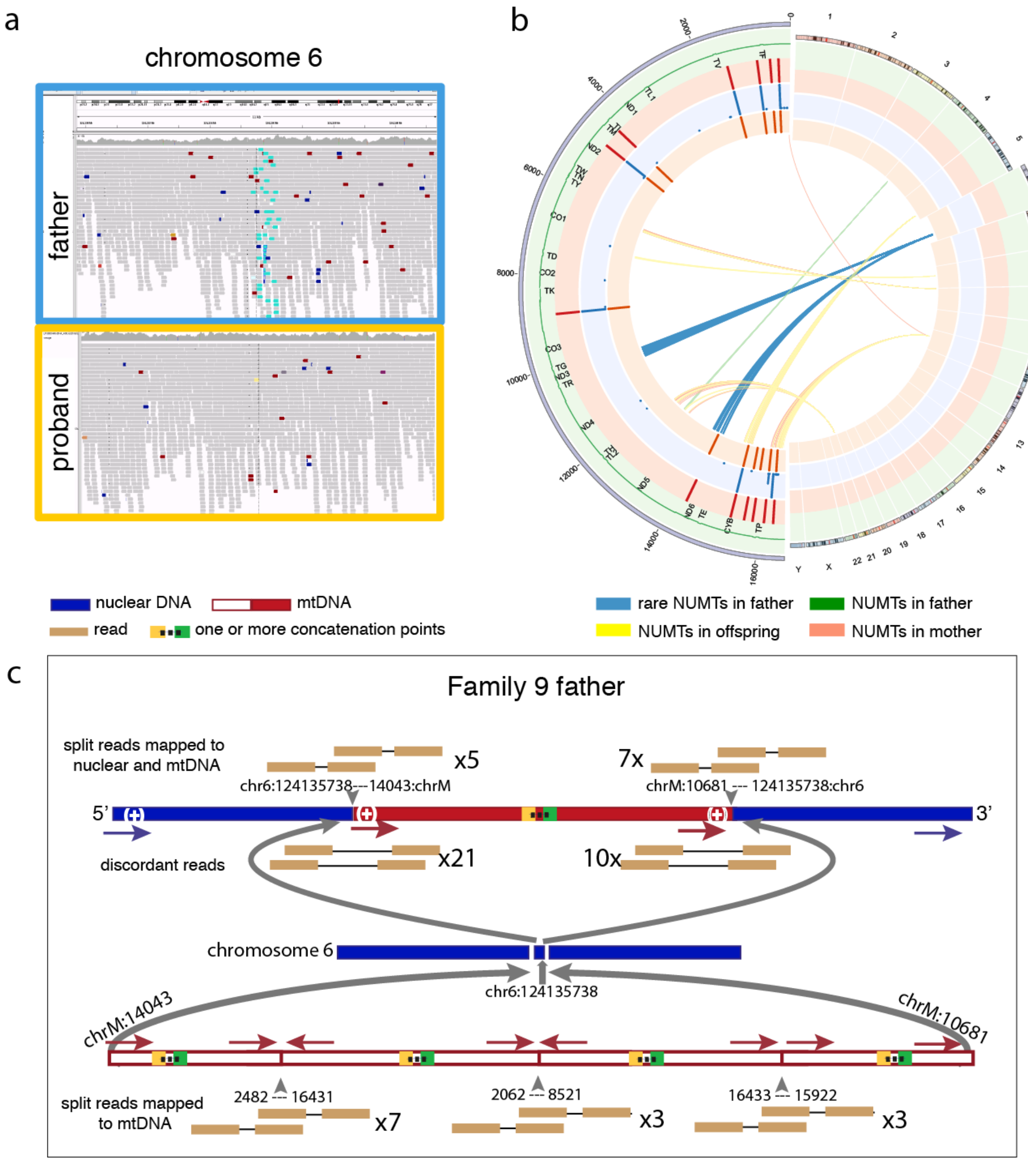

nonTransmitted NUMTs in Family 10

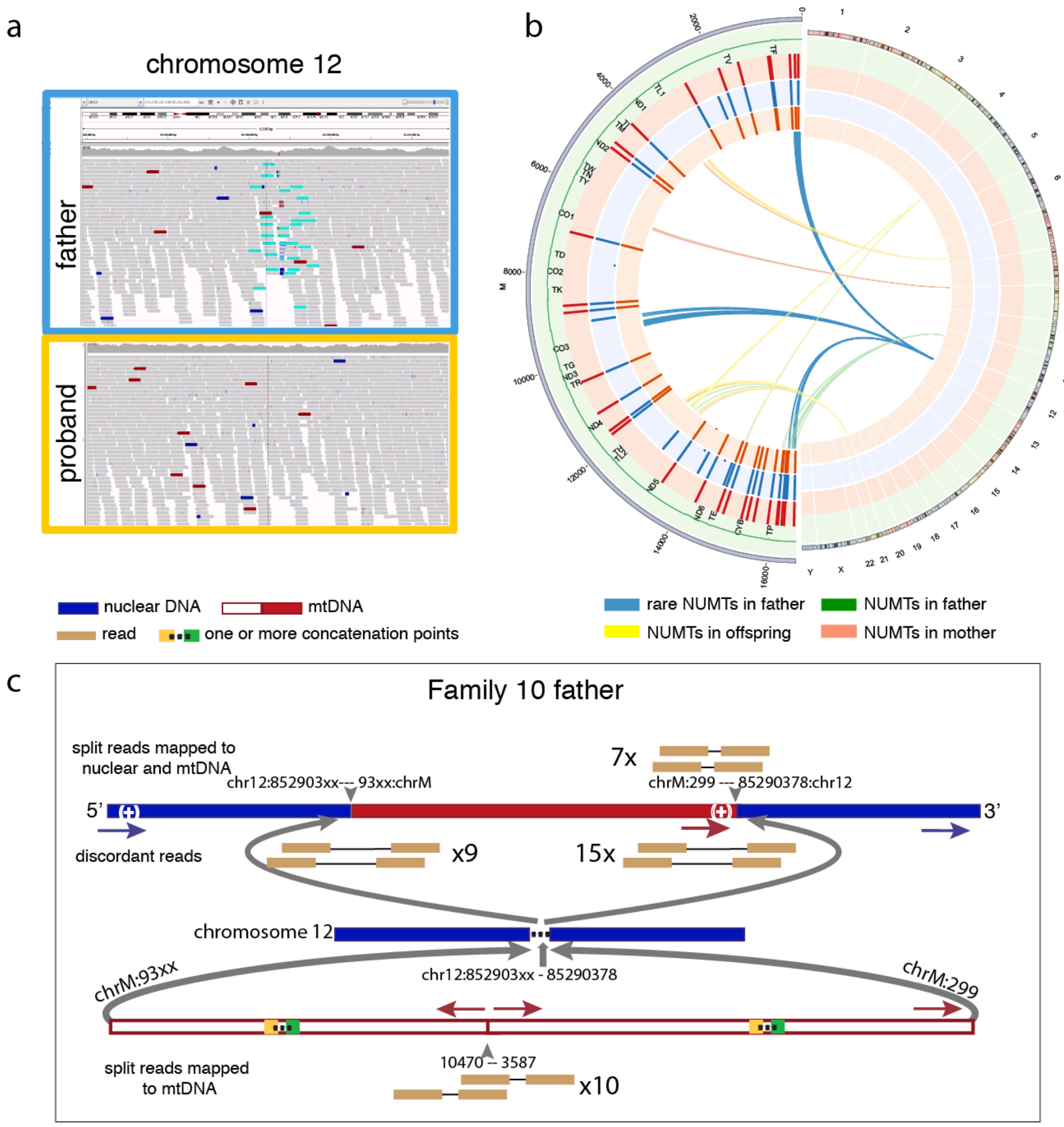

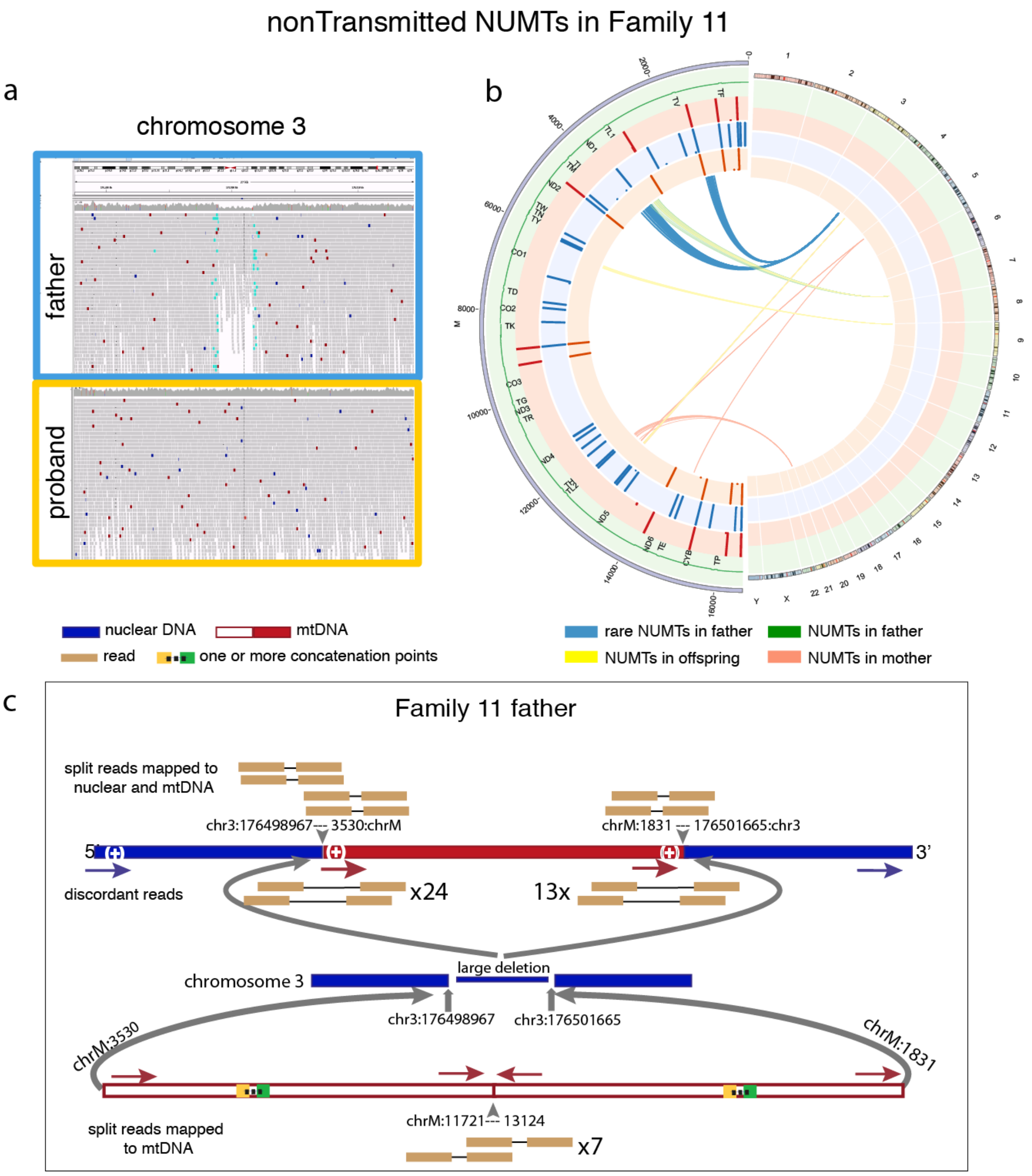
nonTransmitted NUMTs in Family 11
a
chromosome 3
father
proband
b
nuclear DNA
mtDNA
read
one or more concatenation points
rare NUMTs in father
NUMTs in father
NUMTs in offspring
NUMTs in mother
c
Family 11 father
split reads mapped to nuclear and mtDNA
chr3:176498967--- 3530:chrM
chrM:1831 --- 176501665:chr3
5'
3'
discordant reads
x24
13x
large deletion
chromosome 3
chr3:176498967
chr3:176501665
chrM:3530
chrM:1831
split reads mapped to mtDNA
chrM:11721--- 13124
x7

nonTransmitted NUMTs in Family 12

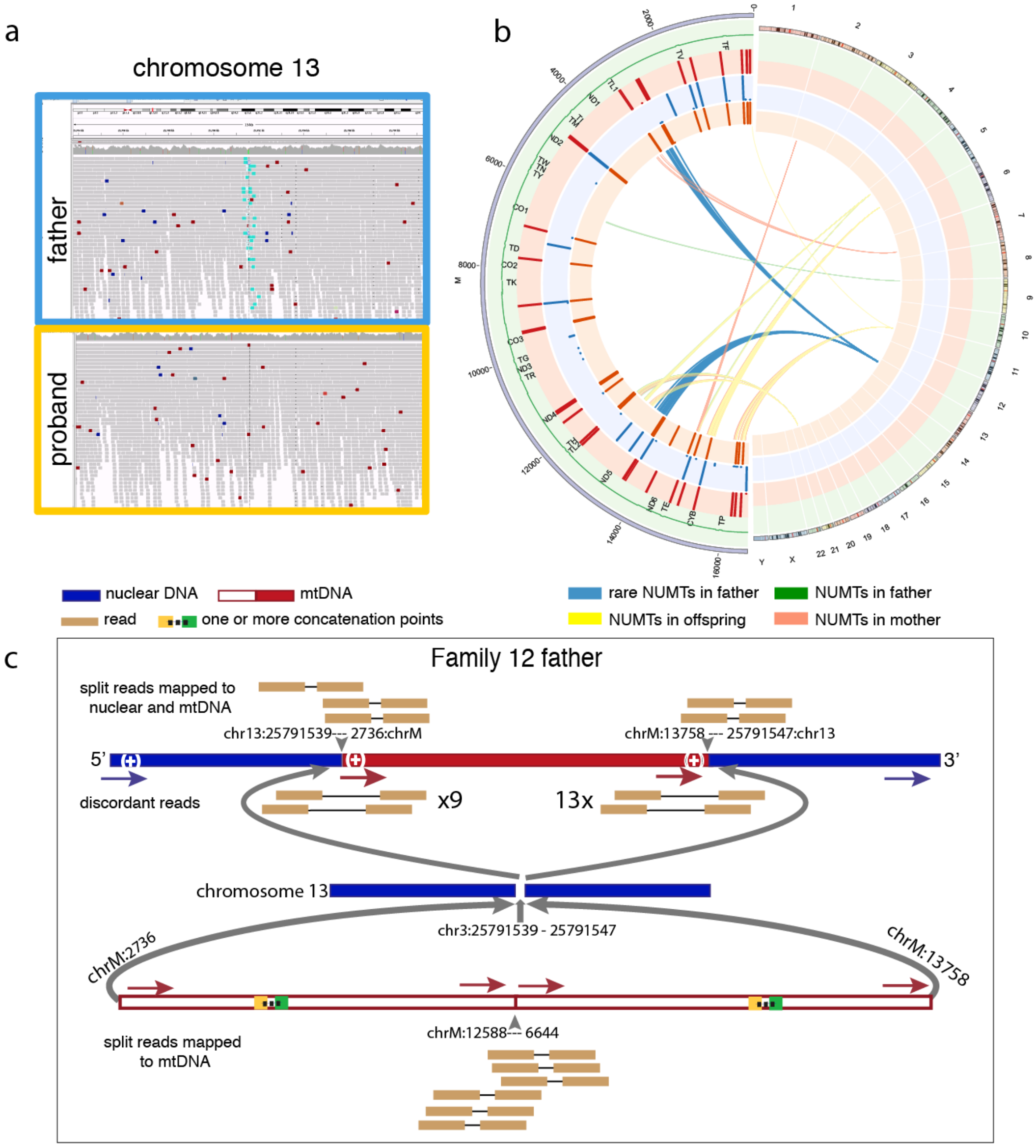

nonTransmitted NUMTs in Family 13

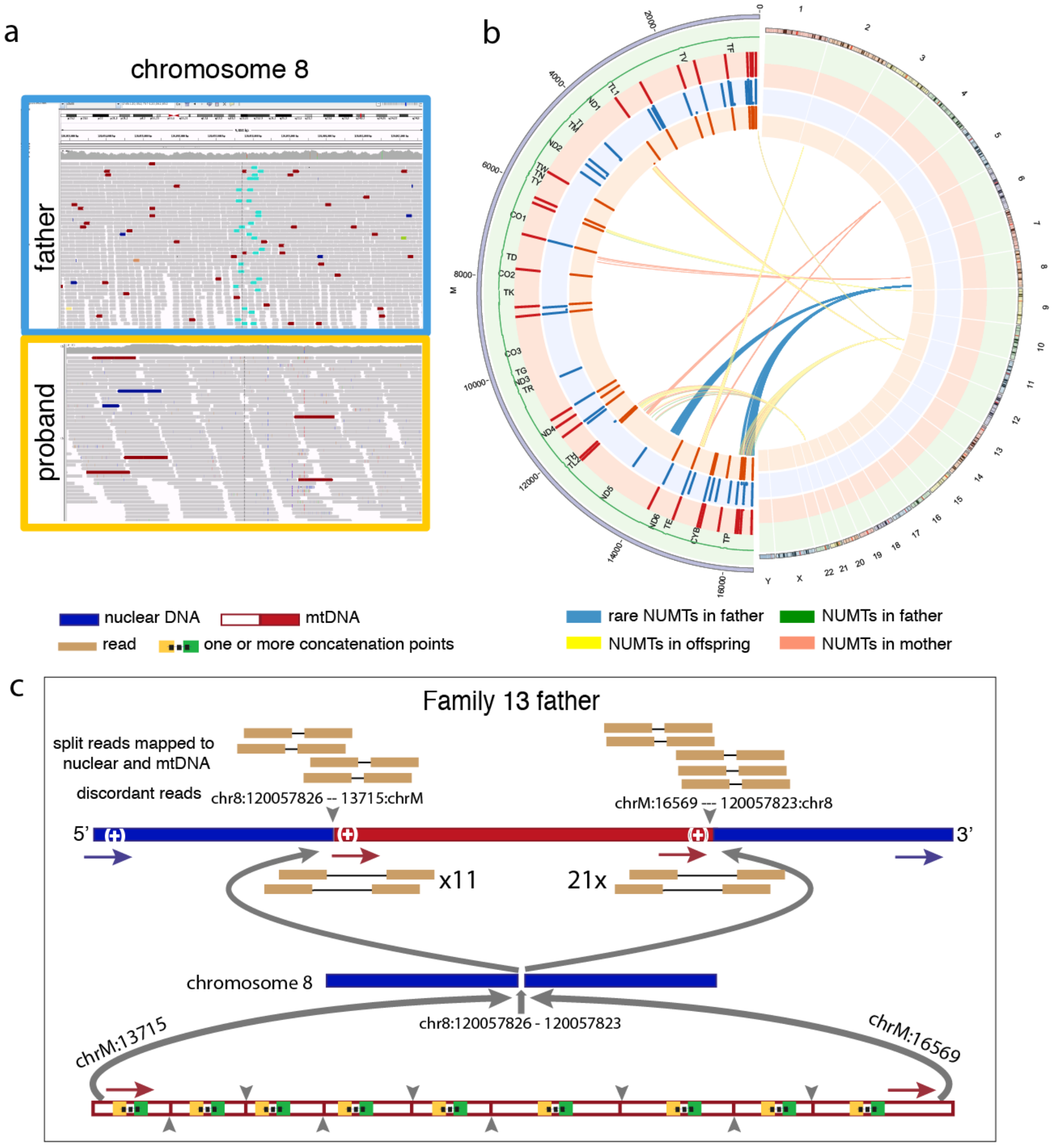

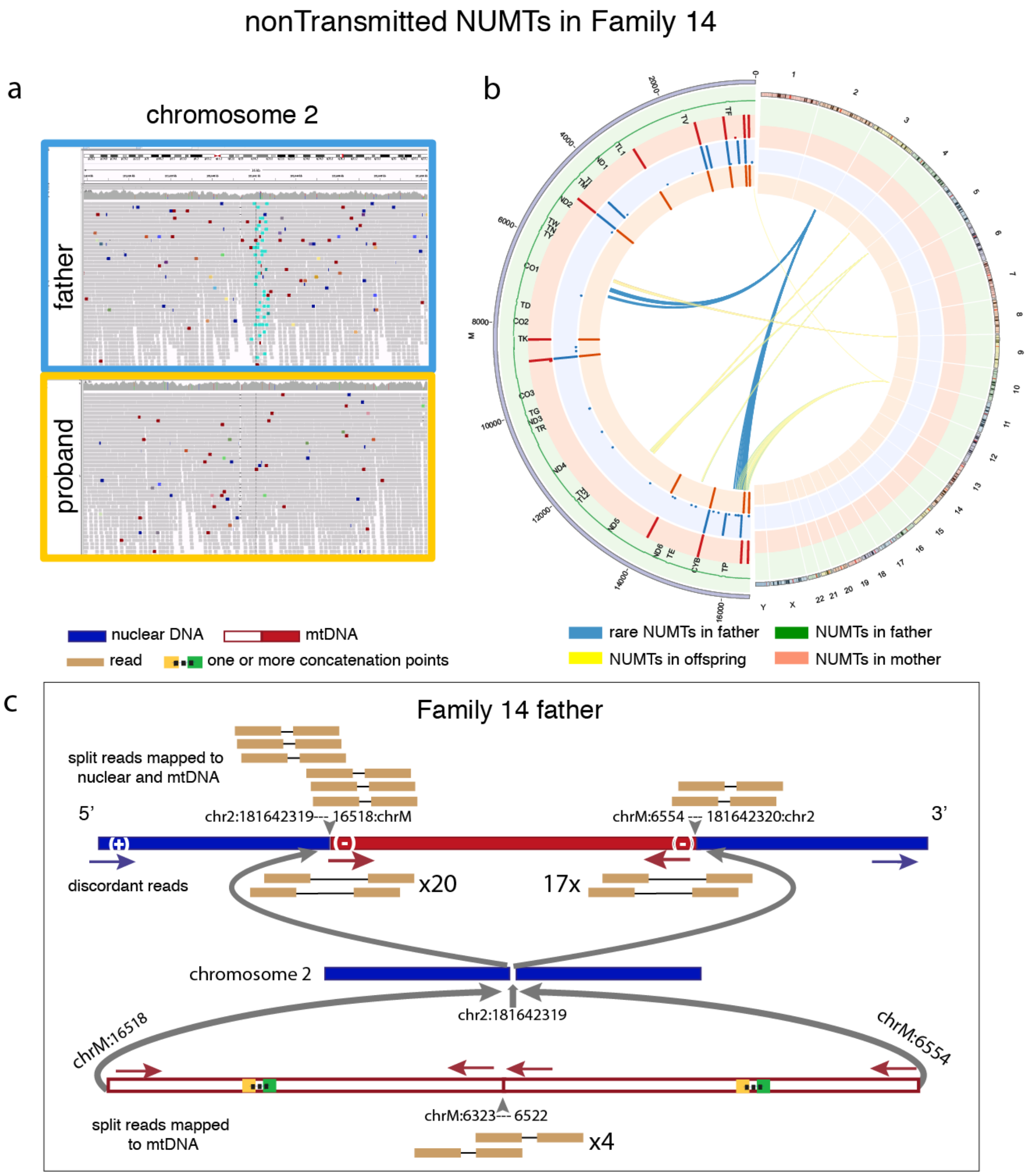
nonTransmitted NUMTs in Family 14
a
chromosome 2
father
proband
b
nuclear DNA
mtDNA
read
one or more concatenation points
rare NUMTs in father
NUMTs in father
NUMTs in offspring
NUMTs in mother
c
Family 14 father
split reads mapped to nuclear and mtDNA
5'
3'
chr2:181642319--- 16518:chrM
chrM:6554 --- 181642320:chr2
discordant reads
x20
17x
chromosome 2
chr2:181642319
chrM:16518
chrM:6554
split reads mapped to mtDNA
chrM:6323--- 6522
x4

Supplementary Figure 7 Variants and NUMTs observed in 6 fathers carrying non-transmitted mega-NUMTs. (a) IGV screenshots show aligned reads corresponding to NUMTs. Teal bars indicate the aligned reads which mapped to the nuclear DNA where their mates mapped to the mtDNA. (**b)** Circos plots shows the detected variants and NUMTs in each family. Circles from the outside to the inside indicate the following: (1) position of a variant on the mtDNA; (2) regions corresponding to the different mtDNA genes; (3) variants identified in the mother where the radial axis corresponds to the AF; (4) variants identified in the father; (5) variants identified in the offspring; (6) NUMTs observed in the family. **(c)** Split reads align to both mtDNA-derived and nuclear DNA sequences (top). Discordant reads are paired reads where one end aligns to mtDNA-derived and the other end aligns to nuclear DNA (middle). Possible constructed concatemer is shown at the bottom with observed supporting split reads. The positions of breakpoints (bp) are shown on both nuclear DNA (top) and mtDNA (bottom).

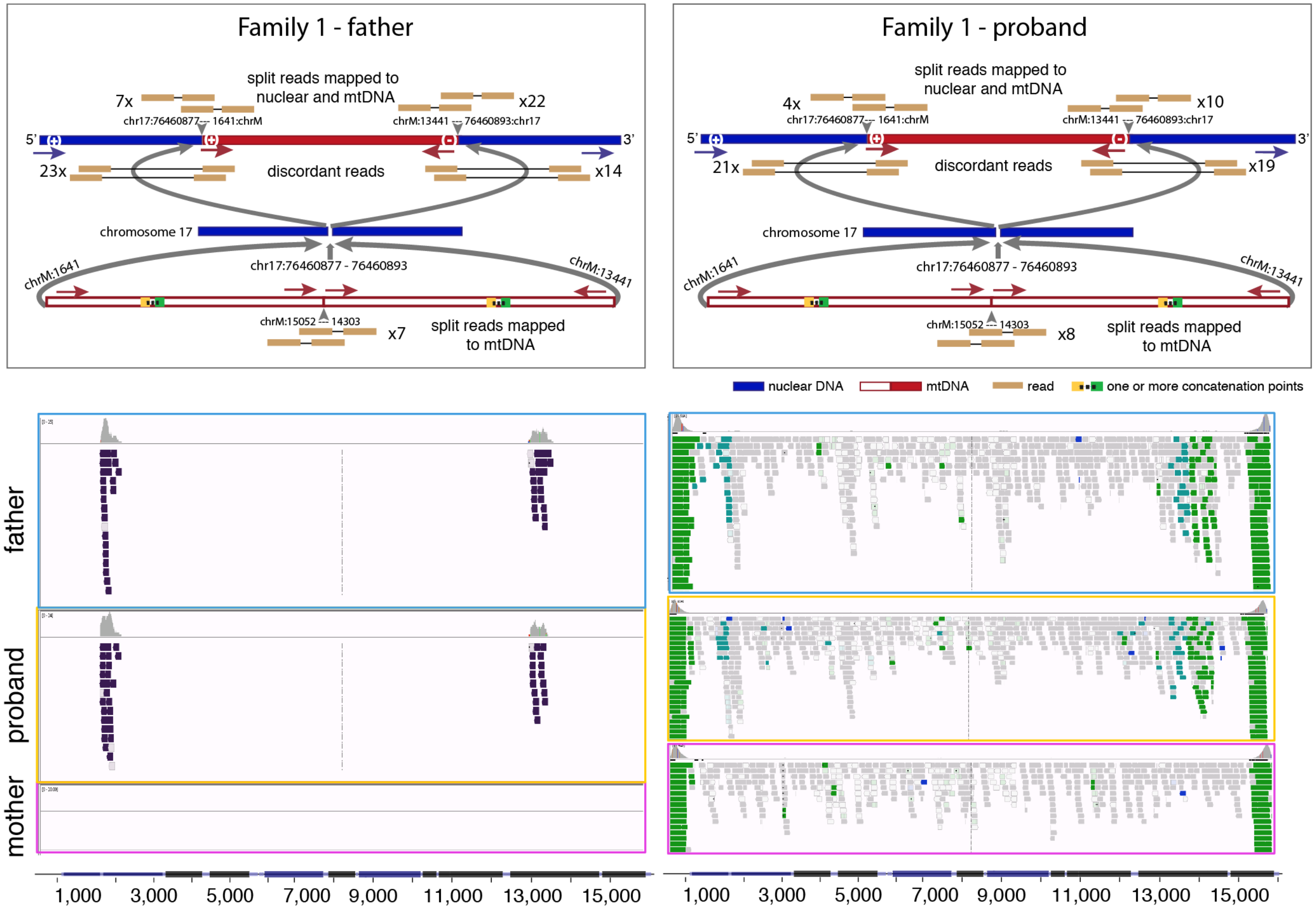

Family 1 - father
split reads mapped to nuclear and mtDNA
7x
x22
chr17:76460877 --- 1641:chrM
chrM:13441 --- 76460893:chr17
5'
3'
23x
discordant reads
x14
chromosome 17
chr17:76460877 - 76460893
chrM:1641
chrM:13441
chrM:15052 --- 14303
x7
split reads mapped to mtDNA
Family 1 - proband
split reads mapped to nuclear and mtDNA
4x
x10
chr17:76460877 --- 1641:chrM
chrM:13441 --- 76460893:chr17
5'
3'
21x
discordant reads
x19
chromosome 17
chr17:76460877 - 76460893
chrM:1641
chrM:13441
chrM:15052 --- 14303
x8
split reads mapped to mtDNA
nuclear DNA
mtDNA
read
one or more concatenation points
father
proband
mother
1,000
3,000
5,000
7,000
9,000
11,000
13,000
15,000

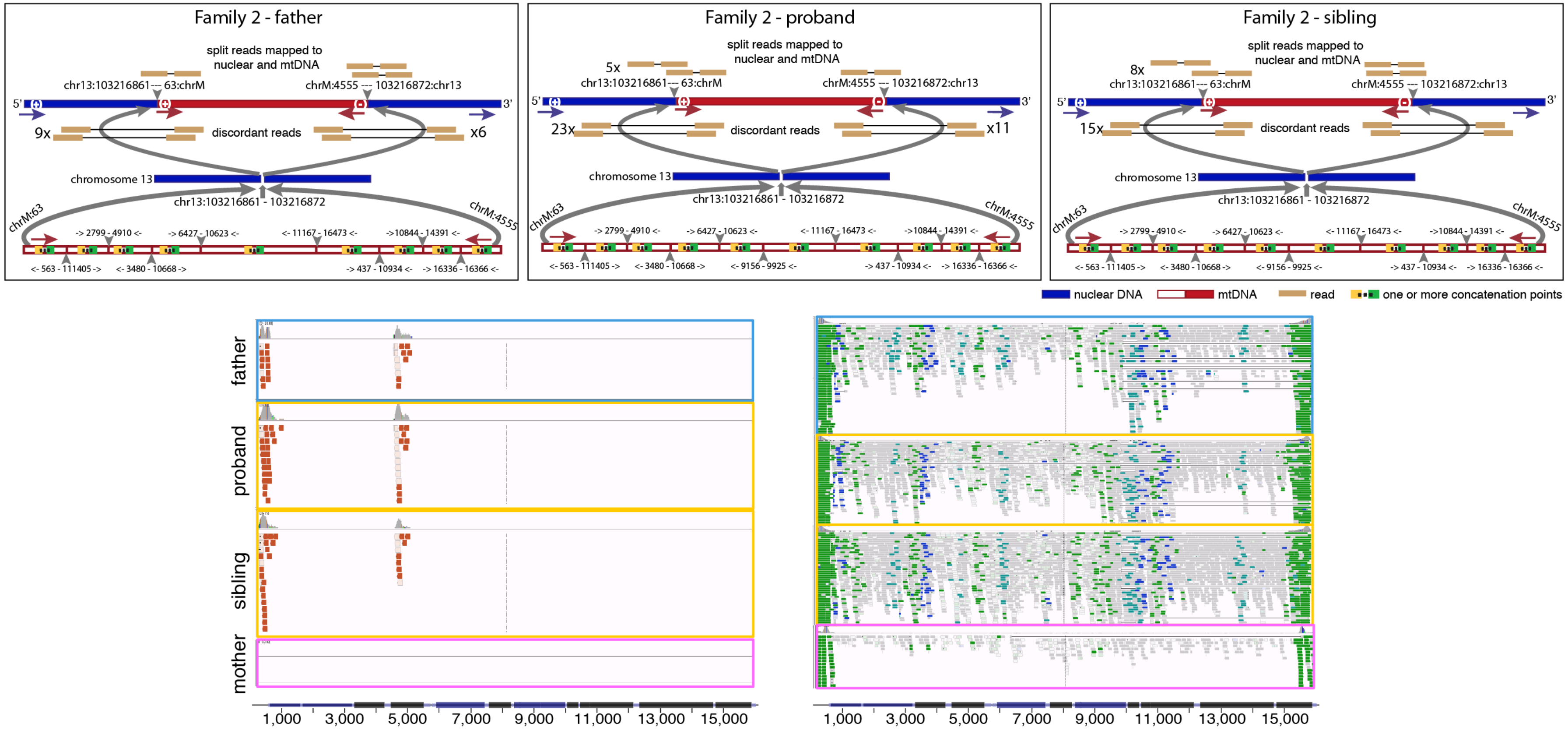

Family 2 - father
Family 2 - proband
Family 2 - sibling
split reads mapped to nuclear and mtDNA
chr13:103216861--- 63:chrM
chrM:4555 --- 103216872:chr13
discordant reads
9x
x6
5x
23x
x11
8x
15x
chromosome 13
chr13:103216861 - 103216872
chrM:63
chrM:4555
5'
3'
nuclear DNA
mtDNA
read
one or more concatenation points
father
proband
sibling
mother
1,000
3,000
5,000
7,000
9,000
11,000
13,000
15,000

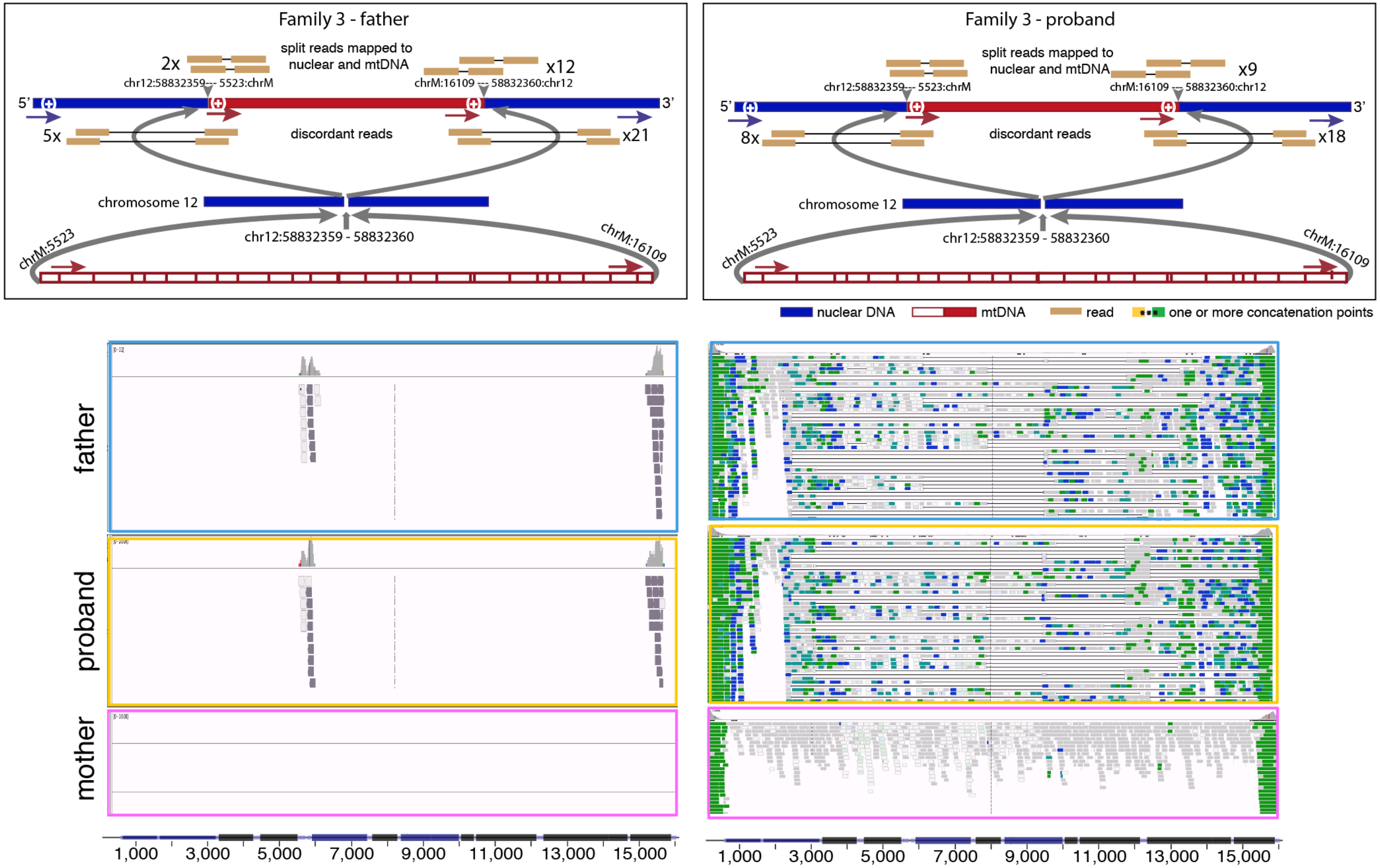

Family 3 - father
split reads mapped to nuclear and mtDNA
2x
x12
chr12:58832359--- 5523:chrM
chrM:16109 --- 58832360:chr12
5'
3'
5x
discordant reads
x21
chromosome 12
chr12:58832359 - 58832360
chrM:5523
chrM:16109
Family 3 - proband
split reads mapped to nuclear and mtDNA
x9
8x
x18
nuclear DNA
mtDNA
read
one or more concatenation points
father
proband
mother
1,000
3,000
5,000
7,000
9,000
11,000
13,000
15,000

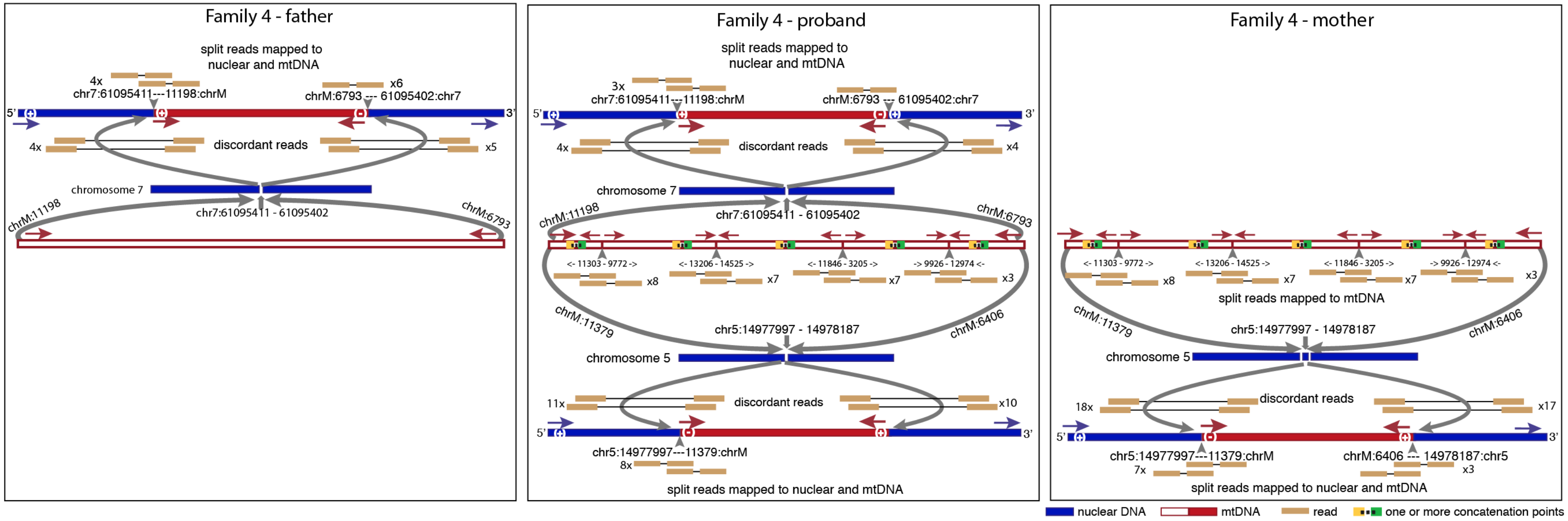
Family 4 - father
split reads mapped to nuclear and mtDNA
4x
chr7:61095411---11198:chrM
x6
chrM:6793 --- 61095402:chr7
4x
discordant reads
x5
chromosome 7
chrM:11198
chr7:61095411 - 61095402
chrM:6793
Family 4 - proband
split reads mapped to nuclear and mtDNA
3x
chr7:61095411---11198:chrM
chrM:6793 --- 61095402:chr7
4x
discordant reads
x4
chromosome 7
chrM:11198
chr7:61095411 - 61095402
chrM:6793
<- 11303 - 9772 ->
x8
<- 13206 - 14525 ->
x7
<- 11846 - 3205 ->
x7
-> 9926 - 12974 <-
x3
chrM:11379
chr5:14977997 - 14978187
chrM:6406
chromosome 5
11x
discordant reads
x10
chr5:14977997---11379:chrM
8x
split reads mapped to nuclear and mtDNA
Family 4 - mother
<- 11303 - 9772 ->
x8
<- 13206 - 14525 ->
x7
<- 11846 - 3205 ->
x7
-> 9926 - 12974 <-
x3
split reads mapped to mtDNA
chrM:11379
chr5:14977997 - 14978187
chrM:6406
chromosome 5
18x
discordant reads
x17
chr5:14977997---11379:chrM
7x
chrM:6406 --- 14978187:chr5
x3
split reads mapped to nuclear and mtDNA
nuclear DNA
mtDNA
read
one or more concatenation points

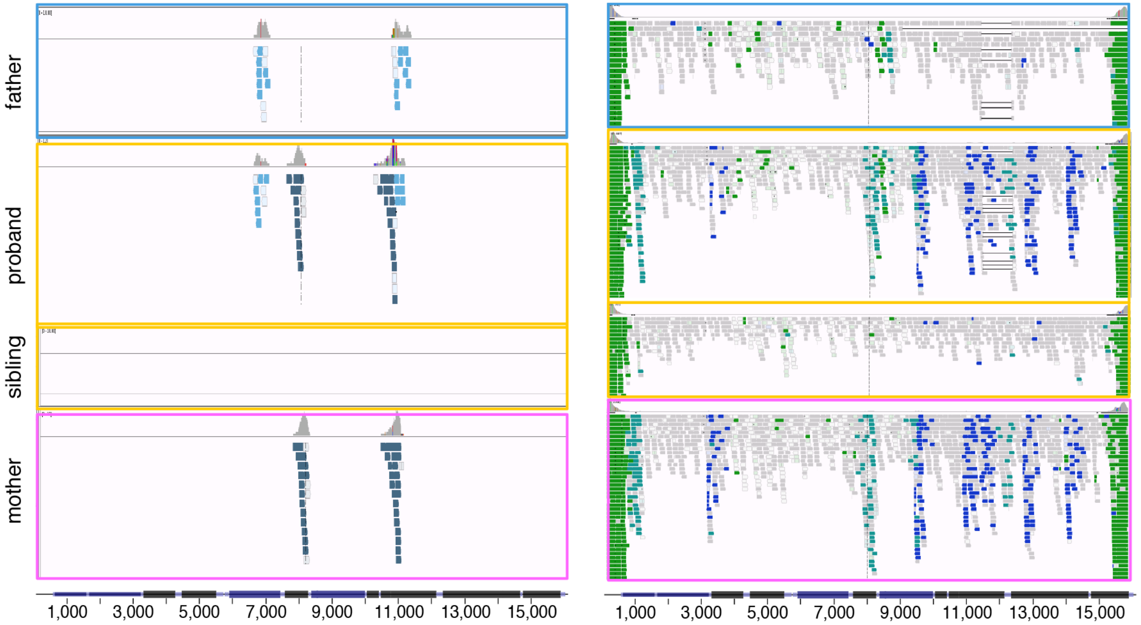
father
proband
sibling
mother
1,000
3,000
5,000
7,000
9,000
11,000
13,000
15,000
1,000
3,000
5,000
7,000
9,000
11,000
13,000
15,000

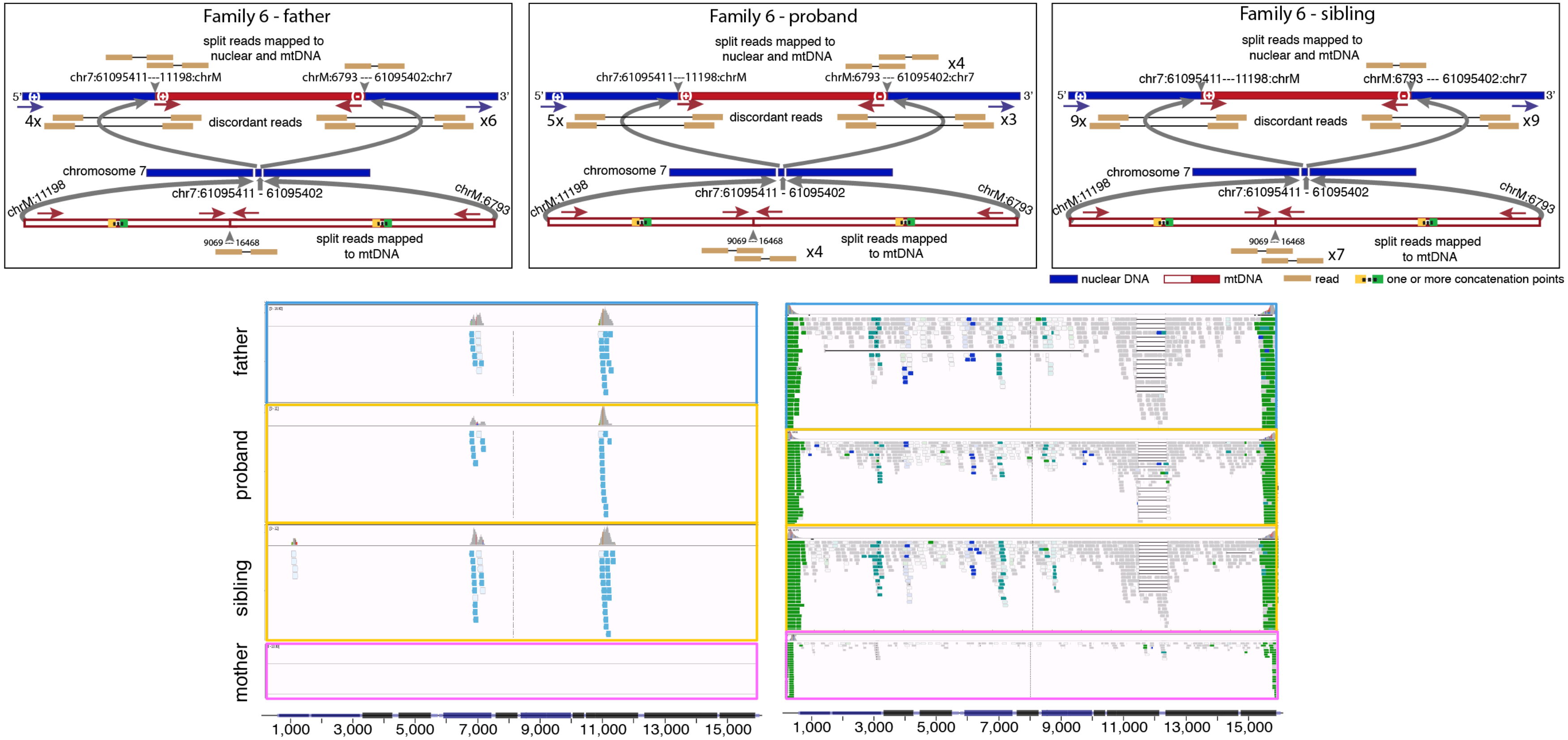

Family 6 - father
split reads mapped to nuclear and mtDNA
chr7:61095411---11198:chrM
chrM:6793 --- 61095402:chr7
4x
discordant reads
x6
chromosome 7
chr7:61095411 - 61095402
chrM:11198
chrM:6793
9069
16468
split reads mapped to mtDNA
Family 6 - proband
split reads mapped to nuclear and mtDNA
x4
5x
x3
x4
Family 6 - sibling
9x
x9
x7
nuclear DNA
mtDNA
read
one or more concatenation points
father
proband
sibling
mother
1,000
3,000
5,000
7,000
9,000
11,000
13,000
15,000

Supplementary Figure 8 Defining mega-NUMTs in 5 families. Within each family, split reads align to both nuclear DNA and mtDNA-derived sequences (top). Discordant reads are paired reads where one end aligns to mtDNA-derived and the other end aligns to nuclear DNA (middle). Possible constructed concatemer is shown at the bottom with observed supporting split reads. The positions of breakpoints (bp) are shown on both nuclear DNA (top) and mtDNA (bottom). (Bottom) Left: IGV screenshots show the alignment of the discordant and split reads corresponding to the mega-NUMTs on the mtDNA-derived sequence. The chromosomes on which their mates are found are shown in different colors. Right: IGV screenshots show the alignment of split reads aligned to mtDNA only. The reads are colored by pair orientation. Many read pairs with anomalous pair orientations in the fathers and probands support the mtDNA-derived rearrangement. In family 4, proband also inherited another mega-NUMT from the mother. Both mother and proband carried the same mtDNA-derived junctions. The mtDNA genome position track from UCSC genome browser is shown at the bottom.

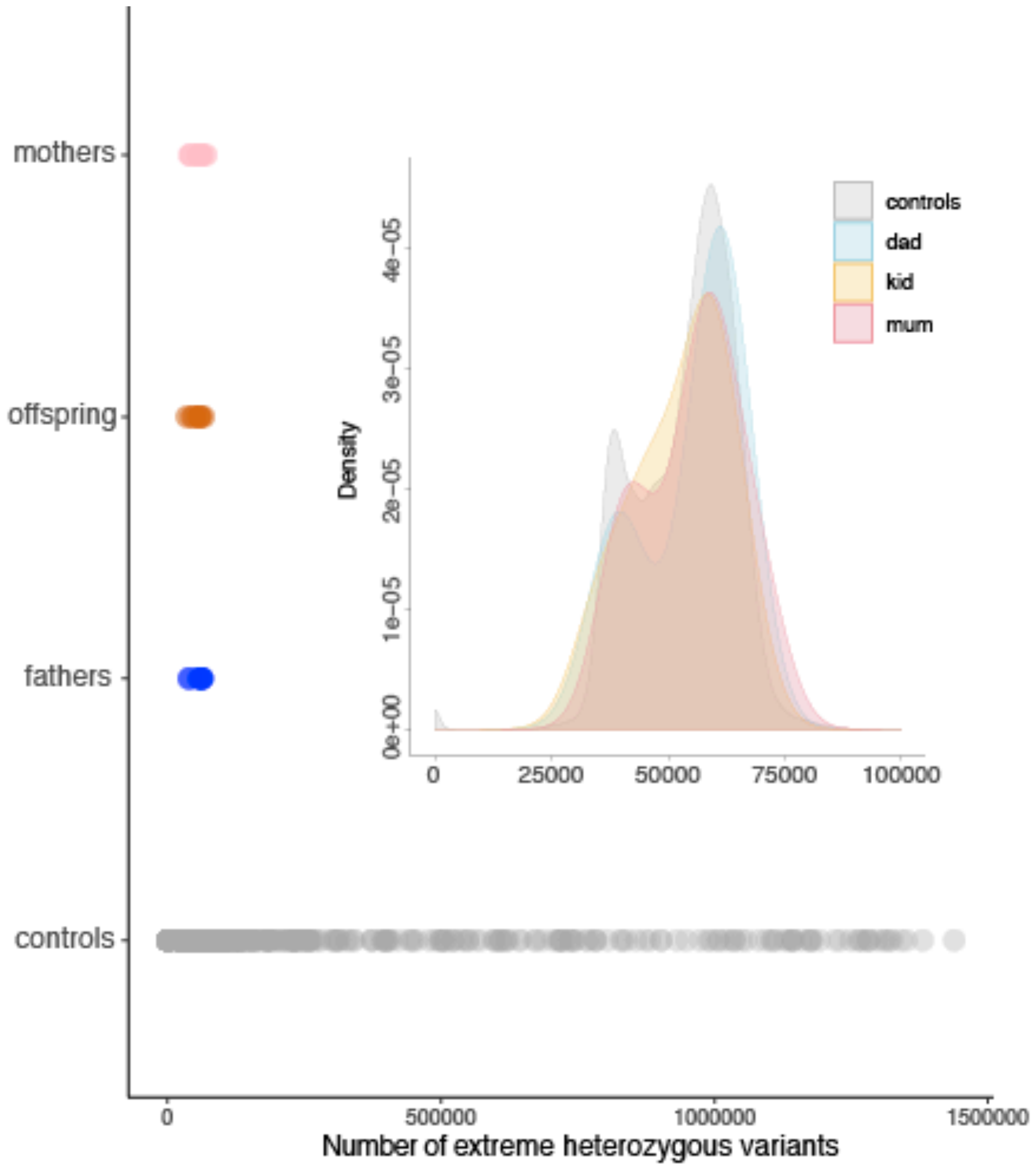

Supplementary Figure 9. Number of extreme heterozygous variants. Father, mother and offspring from 7 families carrying mixed haplotypes are shown in different colors. The numbers of extreme heterozygous variants from the remaining individuals in the whole dataset are shown at the bottom in grey circles. The distributions of number of extreme heterozygous variants from mother, father, offspring in 7 families and the whole dataset are shown in the middle.

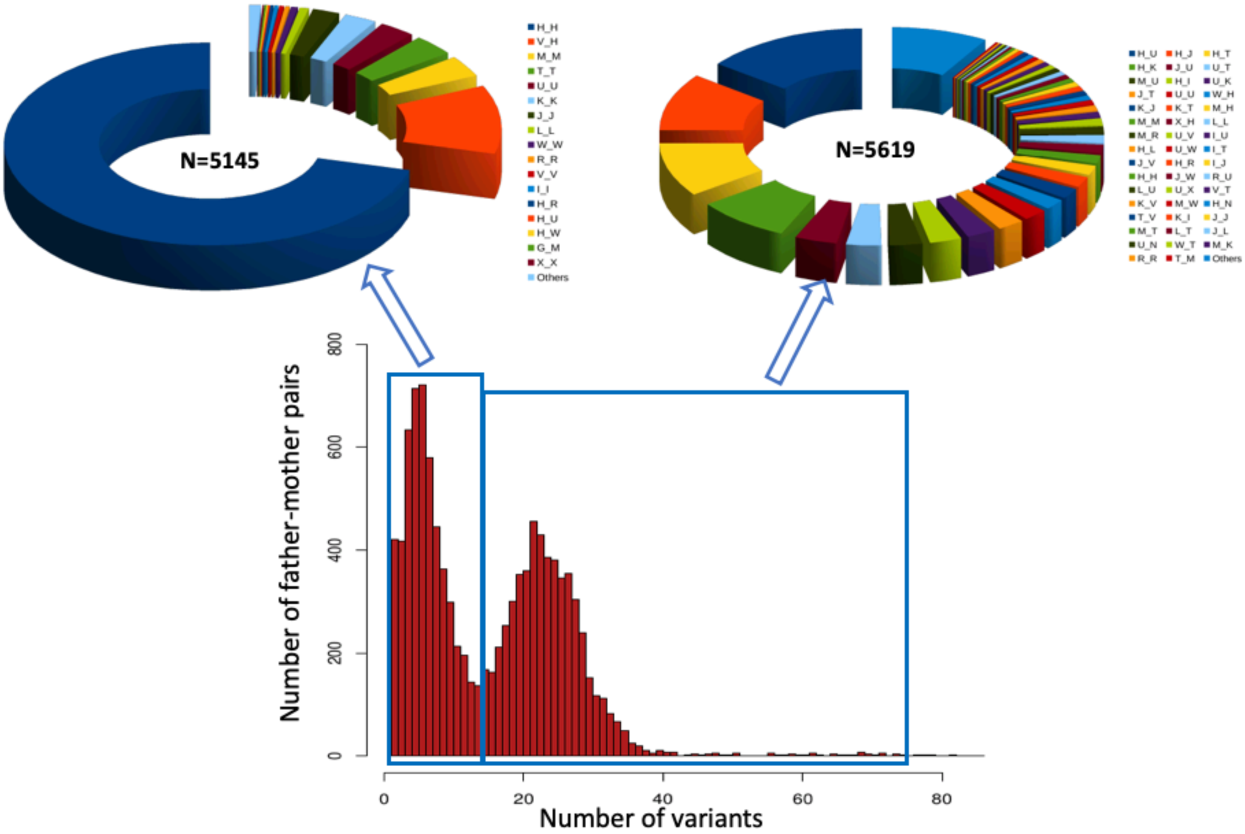

Supplementary Figure 10 Distribution of mitochondrial haplogroups between each father-mother pair. The left pie chart shows the father-mother pairs with fewer than 14 variants which are only present in the fathers. The right pie chart shows the father-mother pairs with at least 14 variants which are only present in the fathers.

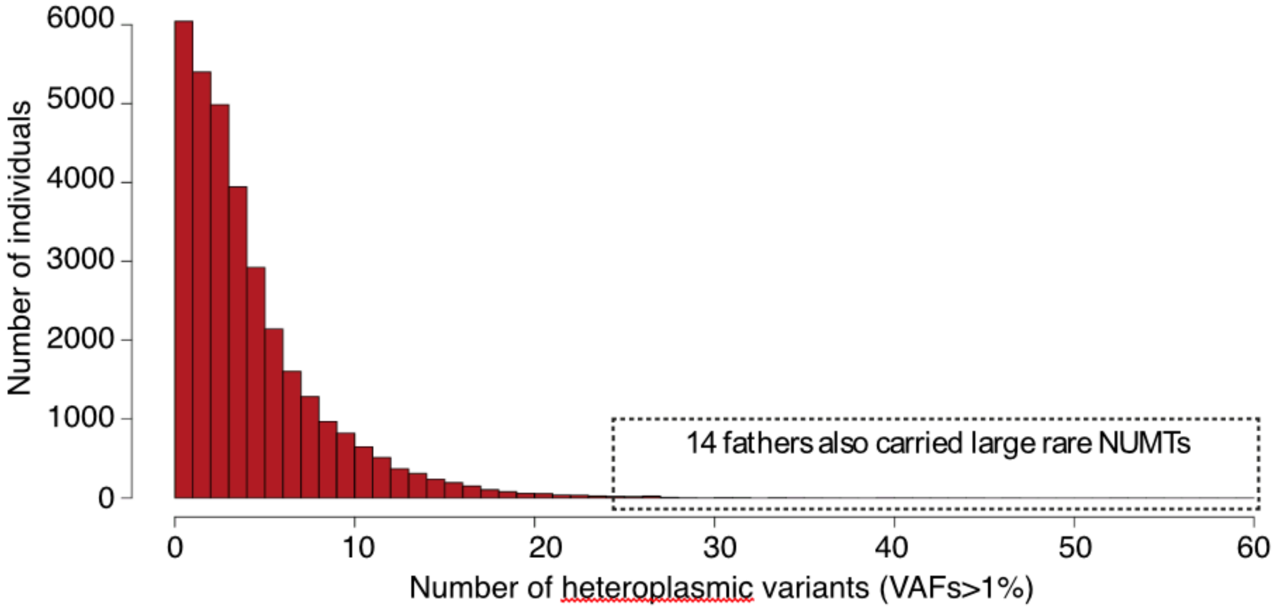

Supplementary Figure 11 Distribution of number of heteroplasmic variants (AFs>1%) per mtDNA sequence. All 14 fathers carrying mega-NUMTs harbored more than 23 heteroplasmic variants.

Supplementary Tables

Supplementary Table 1. Summary of non-transmitted mega-NUMTs in 6 fathers.

FatherID	Junction1 (hg38)		Junction2 (hg38)		Father		
	Nuclear	mtDNA	Nuclear	mtDNA	DCRs	SPRs	mtJunctions
**Family9**	chr6:124135738	10681(+)	chr6:124135738	14043(+)	31	12	3
**Family10**	chr12:852903xx	93xx	chr12:85290378	299(+)	24	7	1
**Family11**	chr3:176498967	3530(+)	chr3:176501665	1831(+)	37	6	1
**Family12**	chr13:25791539	2736(+)	chr13:25791547	13758(+)	22	5	1
**Family13**	chr8:120057826	13715(+)	chr8:120057823	16569(+)	32	9	8
**Family14**	chr2:181642319	16518(-)	chr2:181642320	6554(-)	37	8	1

DCRs = discordant reads; SPRs = split reads.

Supplementary Table 2. Summary of breakpoints within mtDNA-derived fragments.

**Family ID**	**No. of breakpoints within mtDNA-derived fragments**			
	**father**	**proband**	**sibling**	**mother**
Family1	1	1	NA	0
Family2	8	9	9	0
Family3	32	32	NA	0
Family4	0	4	0	4
Family5	3	2	NA	0
Family6	1	1	1	0
Family7	3	3	NA	0
Family8	1	1	NA	0
Family9	3	0	NA	0
Family10	1	0	NA	0
Family11	1	0	NA	0
Family12	1	0	NA	0
Family13	8	0	NA	0
Family14	1	0	NA	0

NA: samples are not available

Supplementary Table 3. Validation of NUMTs observed in short-read sequencing by long-read (Oxford Nanopore PromethION) sequencing.

**NUMTs**	**Junction1 (GRCh37)**		**Junction2 (GRCh37)**		**Length**	**Individual IDs***				
**ID**	**Nuclear**	**mtDNA**	**Nuclear**	**mtDNA**	**(bp)**	**1**	**2**	**3**	**4**	**5**
**1**	2:33892482	15022(-)	2:33892479	14776(-)	246			Y		Y
**2**	2:212977624	10368(-)	2:212977626	9187(-)	1181				Y	
**3**	4:29441247	12997(-)	4:29441248	12834(-)	163	Y	Y		Y	
**4**	4:190598242	12360(+)	-	13227(+)	867	Y	Y			
**5**	5:32338583	14803-14977(+)	5:32338584	12864-12714 (-)	324	Y		Y		Y
**6**	8:63015431	8553(-)	8:63015423	7083(-)	1470		Y	Y	Y	Y
**7**	8:82385249	15834(+)	8:82385262	3108(+)	3843					Y
**8**	9:129775	6420(-)	9:129767	6224(-)	196	Y	Y	Y		
**9**	11:49883569	61(-)	11:49883572	16088(-)	542	Y	Y		Y	Y

* if Y in the cell, NUMT (each row represents a unique NUMT) is observed in an individual (sample IDs are listed in the last 5 columns).

Supplementary Table 4 Estimated number of copies of the mtDNA-derived fragment (No) within the NUMT in 7 families.

Family1				Famliy2						Family3				Family4					
father		proband		father		proband		sibling		father		proband		father		proband		sibling	
POS	No	POS	No	POS	No	POS	No	POS	No	POS	No	POS	No	POS	No	POS	No	POS	No
73	2	189	3	146	9	146	8	146	9	73	3	73	3	152	19	143	21	152	19
150	3	195	3	199	9	827	7	827	8	489	5	489	6	199	19	152	20	199	19
152	3	204	3	489	5	4820	7	4820	9	2706	5	750	8	489	11	373	12	489	11
271	3	207	4	827	8	5465	10	5465	11	3736	6	1438	8	3780	12	1598	14	3780	12
295	2	489	2	3606	8	6719	11	6719	12	3745	6	2706	7	6366	12	3864	14	6366	12
489	2	1243	3	4071	10	7521	8	7521	10	4823	6	3736	7	8450	16	4062	15	8450	16
709	2	2483	3	4820	10	8392	11	8392	11	5786	6	3745	7	8594	15	4225	16	8594	15
2706	3	3398	3	4850	11	9101	9	9101	10	6956	6	4080	7	8701	18	5836	19	8701	18
11719	3	3505	3	5442	12	9123	10	9123	11	7028	5	4754	8	9452	9	7490	16	9452	9
12612	3	5046	4	6455	11	10238	8	10238	9	7621	7	4769	8	9540	14	8584	14	9540	14
13708	2	5460	3	7521	9	11239	8	11239	9	8251	4	4823	7	10398	14	8594	14	10398	14
14766	3	8994	3	8701	11	11914	8	11914	9	8701	6	5786	8	10400	14	8805	14	10400	14
15452	2	11674	3	9101	9	12239	8	12239	10	9058	7	6956	6	10754	13	9174	13	10754	13
16069	2	11947	3	9540	11	13590	8	13590	10	9355	6	7028	5	10873	16	9181	13	10873	16
16126	3	12414	4	9824	11	14221	9	14221	10	9540	7	8251	6	11293	14	9452	8	11293	14
16278	3	12705	3	10398	10	14587	7	14587	10	10398	7	8701	8	11732	18	10631	15	11732	18
16356	4	15884	4	10400	10	15326	8	15326	9	10400	7	9180	8	13635	16	10754	12	13635	16
16519	1	16223	4	10873	9	15535	7	15535	8	10873	5	9540	6	13971	13	11009	10	13971	13
		16292	4	11239	8	15746	10	15746	12	11719	4	10398	8	14040	13	11293	13	14040	13
				11665	9	16136	7	16136	7	12705	7	10400	8	14544	13	12406	16	14544	13
				11914	9	16172	4	16172	5	13145	4	10873	7	14783	13	13236	19	14783	13
				12091	10	16175	4	16175	4	14061	6	11719	6	14990	13	13635	18	14990	13
				12705	12	16218	5	16218	4	14766	6	12705	9	15043	15	13971	10	15043	15
				13590	9	16261	8	16261	8	14783	6	13145	7	15301	13	14040	15	15301	13
				14587	8					15043	7	14061	6	15385	12	14194	17	15385	12
				14783	9					15119	6	14766	7	15900	15	14305	17	15900	15
				15043	10					15301	7	14783	7	16093	28	14544	16	16093	28
				15236	8					15622	7	15043	7	16129	15	14577	18	16129	15
				15301	9					15777	5	15119	7	16223	17	14990	11	16223	17
				15535	6					16223	7	15301	6	16225	17	15385	15	16225	17
				16136	7					16362	8	15622	8	16234	18	15777	20	16234	18
				16223	6					16519	3	16223	9	16266	16	16266	19	16266	16
				16295	9							16235	10	16304	16	16298	19	16304	16
				16324	9							16291	8	16325	17	16299	19	16325	17
				16362	9							16311	8	16356	19	16304	20	16356	19
												16362	7	16392	21	16311	8	16392	21
																16325	12		
																16356	19		
																16456	6		
																16524	4		

Family5				Famliy6						Family7			
father		proband		father		proband		sibling		father		proband	
POS	No	POS	No	POS	No	POS	No	POS	No	POS	No	POS	No
73	5	152	10	143	23	73	11	73	10	73	5	73	5
152	11	709	7	152	22	152	18	152	19	150	12	152	10
195	10	930	7	195	22	195	15	195	19	1811	8	325	5
372	4	3290	7	227	23	961	19	961	17	2294	8	497	6
3197	8	7891	7	337	14	2706	17	2706	17	3010	8	1189	7
3290	8	10321	7	447	17	3145	17	3145	16	3290	8	1811	7
9263	7	10463	8	489	15	7028	18	7028	17	3388	9	3290	8
9477	7	11719	7	1780	21	8448	16	8448	19	4703	9	3480	8
11467	9	13692	6	6647	19	8594	16	8594	17	6518	11	6260	9
11719	9	14233	8	7337	19	9452	14	9452	12	7521	9	9055	10
12308	9	14766	7	8212	18	10754	18	10754	14	9380	9	9698	10
12372	10	14905	7	8502	20	11293	16	11293	13	9698	9	10029	10
13617	8	15452	7	8567	18	11719	16	11719	13	10506	10	10398	10
13827	7	15607	7	8594	20	13635	17	13635	15	11467	9	10550	10
13928	8	15928	7	8701	20	13759	15	13759	16	11719	10	11299	9
14766	9	16126	9	9452	13	13971	15	13971	14	12308	11	11467	10
14793	8	16291	10	9540	19	14040	16	14040	14	12372	12	11485	10
16114	10	16294	10	9899	16	14544	18	14544	15	13934	9	11719	10
16256	11	16296	10	10398	19	14587	18	14587	15	14139	9	11840	9
16270	11	16304	9	10400	20	14766	16	14766	17	14766	9	12308	10
16294	11	16357	8	10754	20	14990	16	14990	16	15454	7	12372	10
16350	11	16497	6	10873	18	15385	15	15385	14	16343	11	13740	8
16357	10	16519	4	11083	18	16092	18	16092	18	16357	10	14167	10
16497	6			11293	16	16140	17	16140	18	16390	11	14766	9
16526	4			11518	19	16266	19	16266	17	16497	6	14798	9
				12705	17	16293	19	16293	18	16519	4	16224	11
				13254	20	16304	21	16304	18			16311	10
				13281	20	16311	19	16311	18			16357	8
				13635	18	16325	18	16325	17			16497	7
				13708	17	16356	17	16356	17			16519	5
				13971	18								
				14040	17								
				14544	18								
				14783	19								
				14861	18								
				14990	16								
				15043	17								
				15253	17								
				15301	16								
				15385	15								
				15670	20								
				16129	17								
				16223	21								
				16266	19								
				16274	21								
				16294	22								
				16304	20								
				16319	22								
				16320	21								
				16325	20								
				16356	20								
				16362	21								
				16518	12								

Supplementary Table 5. Sequencing depth of 7 families where father and offspring carrying the mixed haplotypes.

Group	relatedness	mean mtDNA depth (x)	mean WGS depth (x)
Family1	father	711	39
Family1	proband	1106	48
Family1	mother	717	38
Family2	father	1702	35
Family2	proband	2474	39
Family2	sibling	4086	52
Family2	mother	1357	37
Family3	father	1368	43
Family3	proband	2235	44
Family3	mother	1966	39
Family4	father	991	38
Family4	proband	1308	36
Family4	sibling	1022	42
Family4	mother	1312	38
Family5	father	1627	35
Family5	proband	2397	35
Family5	mother	1092	35
Family6	father	1046	42
Family6	proband	1315	43
Family6	sibling	1041	42
Family6	mother	1091	43
Family7	father	2389	40
Family7	proband	2609	40
Family7	mother	2436	42
