# Supplementary material for: Nuclear-mitochondrial DNA segments resemble paternally inherited mitochondrial DNA in humans
Source: Nat Commun. 2020 Apr 8;11:1740. doi: 10.1038/s41467-020-15336-3 (PMC7142097; doi:10.1038/s41467-020-15336-3)
Supplement: Supplementary file 2 — Reporting Summary [file 41467_2020_15336_MOESM2_ESM.pdf]

nature research

Corresponding author(s): NCOMMS-19-23131

Last updated by author(s): Jul 10, 2019

Reporting Summary

Nature Research wishes to improve the reproducibility of the work that we publish. This form provides structure for consistency and transparency in reporting. For further information on Nature Research policies, see Authors & Referees and the Editorial Policy Checklist .

Statistics

For all statistical analyses, confirm that the following items are present in the figure legend, table legend, main text, or Methods section.

n/a	Confirmed	
☐	☒	The exact sample size (n) for each experimental group/condition, given as a discrete number and unit of measurement
☒	☐	A statement on whether measurements were taken from distinct samples or whether the same sample was measured repeatedly
☐	☒	The statistical test(s) used AND whether they are one- or two-sided<br>*Only common tests should be described solely by name; describe more complex techniques in the Methods section.*
☒	☐	A description of all covariates tested
☐	☒	A description of any assumptions or corrections, such as tests of normality and adjustment for multiple comparisons
☐	☒	A full description of the statistical parameters including central tendency (e.g. means) or other basic estimates (e.g. regression coefficient) AND variation (e.g. standard deviation) or associated estimates of uncertainty (e.g. confidence intervals)
☐	☒	For null hypothesis testing, the test statistic (e.g. F, t, r) with confidence intervals, effect sizes, degrees of freedom and P value noted<br>*Give P values as exact values whenever suitable.*
☒	☐	For Bayesian analysis, information on the choice of priors and Markov chain Monte Carlo settings
☒	☐	For hierarchical and complex designs, identification of the appropriate level for tests and full reporting of outcomes
☐	☒	Estimates of effect sizes (e.g. Cohen's d, Pearson's r), indicating how they were calculated

Our web collection on statistics for biologists contains articles on many of the points above.

Software and code

Policy information about availability of computer code

Data collection	The software and data used in this study are all cited in the manuscript.
Data analysis	The software and methods used to do the analysis are all cited and described in the manuscript.

For manuscripts utilizing custom algorithms or software that are central to the research but not yet described in published literature, software must be made available to editors/reviewers. We strongly encourage code deposition in a community repository (e.g. GitHub). See the Nature Research guidelines for submitting code & software for further information.

Data

Policy information about availability of data

All manuscripts must include a data availability statement. This statement should provide the following information, where applicable:

- Accession codes, unique identifiers, or web links for publicly available datasets
- A list of figures that have associated raw data
- A description of any restrictions on data availability

The sequence data will be available for analysis through the Genomics England data warehouse.

Field-specific reporting

Please select the one below that is the best fit for your research. If you are not sure, read the appropriate sections before making your selection.

☒ Life sciences ☐ Behavioural & social sciences ☐ Ecological, evolutionary & environmental sciences

For a reference copy of the document with all sections, see nature.com/documents/nr-reporting-summary-flat.pdf

Life sciences study design

All studies must disclose on these points even when the disclosure is negative.

Sample size	We collected all the trios which whole genome sequence (WGS) data is available in the Genomics England 100,000 Genomes Rare Disease Main Programme at the time we performed the analysis.
Data exclusions	We excluded 832 trios from the main analysis after several quality controls steps. The details were described in the manuscript.
Replication	No replication in other dataset has yet been attempted.
Randomization	Not applicable
Blinding	Not applicable

Reporting for specific materials, systems and methods

We require information from authors about some types of materials, experimental systems and methods used in many studies. Here, indicate whether each material, system or method listed is relevant to your study. If you are not sure if a list item applies to your research, read the appropriate section before selecting a response.

Materials & experimental systems

n/a	Involved in the study	
☒	☐	Antibodies
☒	☐	Eukaryotic cell lines
☒	☐	Palaeontology
☒	☐	Animals and other organisms
☐	☒	Human research participants
☒	☐	Clinical data

Methods

n/a	Involved in the study	
☒	☐	ChIP-seq
☒	☐	Flow cytometry
☒	☐	MRI-based neuroimaging

Human research participants

Policy information about studies involving human research participants

Population characteristics	11, 867 self reported trios in the Genomics England 100,000 Genomes Rare Disease Main Programme were initially analyzed in this study.
Recruitment	Thee Genomics England 100,000 Genomes Rare Disease project enrolled people with a high likelihood or clear evidence of a rare inherited disorder. For the purpose of this study, no any clinical data was included.
Ethics oversight	Ethical approval was provided by the East of England Cambridge South national research ethics committee under reference number: 13/EE/0325, with participants providing written informed consent for this approved study. All consenting participants in the Rare Disease arm of the 100,000 Genomes Project were enrolled via thirteen centres in the National Health Service covering all NHS patients in England.

Note that full information on the approval of the study protocol must also be provided in the manuscript.
